# TME-targeting theranostic agent uses NIR tracking for tumor diagnosis and surgical resection and acts as chemotherapeutic showing enhanced efficiency and minimal toxicity

**DOI:** 10.7150/thno.68074

**Published:** 2022-02-28

**Authors:** Zhongyuan Xu, Jianqiang Qian, Chi Meng, Yun Liu, Qian Ding, Hongmei Wu, Peng Li, Fansheng Ran, Gong-Qing Liu, Yunyun Wang, Yong Ling

**Affiliations:** 1 School of Pharmacy and Jiangsu Province Key Laboratory for Inflammation and Molecular Drug Target, Nantong University, Nantong 226001, PR China.; 2 The Affiliated Hospital of Nantong University, Nantong University, Nantong 226001, PR China.

**Keywords:** Tumor microenvironment, NIR fluorescence, ROS/GSH -dual-responsive, Podophyllotoxin (PPT), Theranostic agent.

## Abstract

**Rationale:** Precise diagnosis and effective therapy of the tumor microenvironment (TME) remains a challenge. Fluorescence tracers for monitoring primary tumors are currently reported; however, they face challenges in accurately delineating tumors in real-time during surgery, including interference from the background and insufficient accumulation of imaging reagents at tumor sites. Additionally, although the natural product podophyllotoxin (PPT) had potent and broad anti-tumor activity, the poor tumor target specificity and high toxicity of PPT extremely limited its clinical application.

**Methods:** In the current study, a novel theranostic agent **PBB** was designed and synthesized by coupling the natural chemotherapeutic drug PPT with a near-infrared (NIR) fluorescence probe hemicyanine (CyOH) via redox-responsive thiolactate linker and introducing biotin to CyOH to enhance the active target ability. The activation mechanism of **PBB** was characterized by absorption spectra, fluorescence spectra, and HPLC. Subsequently, we investigated its imaging action, anti-tumor activity, and toxicity *in vitro* and* in vivo*.

**Results:** *In vitro* experiments, **PBB** was verified to possess a ROS/GSH-responsive molecular switch, impelling **PBB** to release a fluorescent fragment and active drug PPT and selectively lighting up tumor cells but not the normal cells. As such, **PBB** was demonstrated to selectively inhibit the growth of tumor cells by inducing intracellular accumulation of ROS and MMP depolarization. More importantly, **PBB** significantly suppressed hepatic tumor growth and minimized the adverse effects caused by PPT, including acute toxicity and impaired liver function. Finally, the NIR fluorescence accumulated in the tumor tissue and stayed continuous for over 24h, and **PBB** provided precise visualization and highly selective fluorescence diagnosis to guide tumor resection.

**Conclusions:** Therefore, the multilevel targeting theranostic agent provided a novel tool for precise diagnosis, real-time monitoring, and efficient tumor chemotherapy with high safety.

## Introduction

The tumor microenvironment (TME) is a unique heterogeneous environment formed by the interaction between tumor cells and the surrounding environment during tumor cell growth 1-2. The malignant proliferation of tumor cells makes the surrounding microenvironment complex and changeable, which is closely related to the occurrence and development of tumors 3-4. Compared with normal tissues, increased oxidative stress is observed in TME, caused by excessive production of reactive oxygen species (ROS) in the tumor cells under hypoxic conditions 5. The overproduction of ROS induces tumor cells to produce a large amount of glutathione (GSH) in order to balance oxidative stress 6. The concentration of GSH can be as high as 2-10 mM, which is almost 100-1000 times that of the normal tissue extracellular fluid and blood environment (2-20 μM) 7-9. The heterogeneity in TME may manifest in terms of potential redox differences. In addition, tumor progression needs more vitamins, minerals, and other supplements for its survival. Biotin (vitamin H or B7) is a growth promoter and is required for the rapid growth of cancer cells. The biotin-specific receptors are overexpressed on the cancer cell surface, resulting in higher biotin content in tumor cells than the normal cells 10-12. Therefore, effectively combining high-level ROS/GSH response and the characteristics of the targets of biotin to build a multilevel targeting system for theranostic agents 13 will endow them with the capacity to accumulate at the tumor site in higher concentrations and help with accurate diagnosis and therapy for tumors, thus, becoming a promising way to cure carcinoma effectively.

Natural products possess abundant resources for new anti-cancer drug development due to their diverse chemical structures and potent biological activities 14-16. Podophyllotoxin (PPT) is a natural aryl tetrahydrolignin isolated from the plant *Podophyllum* 17. Recent studies have shown that PPT has potent anti-tumor activities and a broader anti-tumor spectrum 18. Its anti-tumor activities mainly prevent tubulin from being assembled into microtubules and induce apoptosis 19. However, PPT has poor tumor target specificity and high toxicity to non-cancerous cells, which hinder its clinical application 20-21. Therefore, it is necessary to adopt multilevel targeting and prodrug strategies 22 to modify the structure of PPT to improve its target specificity, minimize its adverse effects, and improve its clinical therapeutic efficacy.

Among the multiple diagnostic techniques, fluorescence imaging has the capability of accurate cancer diagnosis through non-invasive, real-time, and high-resolution imaging 23-25. In particular, near-infrared (NIR) fluorescence imaging offers deeper tissue penetration depth, weak background interference, and outstanding imaging effectiveness, making it the technique of choice for biomedical applications 25-27. Therefore, NIR fluorescence imaging and targeted therapy were integrated into a single theranostic agent using a clever design to enable simultaneous *in vivo* diagnostic imaging and chemotherapy, and ultimately enhance the efficiency of cancer therapy.

In the current study, we combined natural chemotherapeutic drug PPT and NIR fluorescence probe hemicyanine (CyOH) 28 through redox-responsive linker thiolactate, subsequently introduced biotin receptor-targeting unit to CyOH, and ultimately designed and synthesized a novel multilevel targeting theranostic agent **PBB**. Since **PBB** incorporated multiple potential critical units into a single construct, it offered benefits like a cancer-selective prodrug, "off-on" fluorescence ability, and tumor targeting potency. The constructed biotin moiety specifically targeted the overexpressed biotin receptor on cancer cells, and the thiolactate linker was specifically cleaved by high levels of TME triggers GSH and ROS, further selectively releasing PPT and NIR fluorescence in the tumor tissue. The released fluorescent fragment then induced fluorescence imaging function, which helped us precisely trace tumors and guide surgical resection. In addition, the target-released PPT improved the biodistribution effects, enhanced tumor inhibition, and reduced the toxicity of PPT *in vivo.*

## Materials and Methods

### Materials

All chemical reagents were purchased from commercial sources and used without further purification. The purity of the compounds was analyzed using HPLC (Waters 1525 binary HPLC pump with a Waters 2998 photodiode array detector) on an XBridge-C18 analytic column (5 μm, 4.6 mm × 150 mm). Mass spectra were recorded using a Mariner mass spectrometer (ESI). ^1^H and ^13^C NMR spectra of the tested compounds were obtained using a 400 -MHz Bruker AV spectrometer with deuterated chloroform (CDCl_3_) as the solvent and TMS as the internal standard. Fluorescence spectra were obtained using a fluorescence spectrophotometer (RF-5301PC, Shimadzu, Japan).

### GSH- and ROS-Dependent Emission Spectra

The stock solution of **PBB** was diluted in deionized water (containing 5% v/v DMSO), and 10 mM H_2_O_2_ and 125 μM GSH were added at room temperature to obtain **PBB**- H_2_O_2_ and **PBB**-GSH mixtures, respectively. After particular time intervals (0, 1, 2, 4, 8, 10, 12, and 24 h), the emission spectra were recorded using the fluorescence spectrometer (RF-5301PC). The emission wavelength ranged from 640 nm to 800 nm with excitation at 635 nm.

### Fluorescence Evaluation

**PBB** and the corresponding biological analytes (H_2_O_2_, GSH, Na^+^, K^+^, Cu^2+^, Fe^2+^, Mg^2+^, VcNa, Mn^2+^, ClO^-^, nitroreductase, and NQO1) were diluted in deionized water (containing 5% v/v DMSO) and mixed at room temperature for 30 min. The emission spectra were recorded using the emission wavelength ranging from 640 nm to 800 nm and excitation wavelength of 635 nm.

### Cell Culture and Animal Models

Four cancer cell lines (MCF-7, HepG2, Hela, and HT29) and two normal cell lines (LO2 and CCD841) were cultured in RPMI-1640 medium or DMEM medium with 10 % fetal bovine serum and 1% penicillin-streptomycin solution (100X) at 37 °C in an incubator containing 5% CO_2_. When 70-80% confluence was achieved, the cells were digested using 0.25% trypsin and sub-cultured every 3-4 days.

All animal experimental protocols were approved by Animal Research and Care Committee of the Nantong University. BALB/c mice (female nude mice, 5-6 weeks old) were purchased from the Model Animal Research Center Affiliated (MARCA) of the Nanjing University to establish a subcutaneous tumor model. HepG2 cells (1 × 10^6^) were subcutaneously injected into the mice to establish the tumor models.

### Intracellular Fluorescence Activation

The cell selective fluorescent imaging was studied on the HepG2 and LO2 cells using confocal microscopy images. HepG2 and LO2 cells were seeded in 35 -mm cell culture plates with a density of 2 × 10^5^ cells/well. The cells were cultured in DMEM containing 1 μM** PBB**. After 30 min of incubation, the old medium was removed, and the cells were washed with PBS thrice. Then, the fluorescence images were recorded using the Leica TCS SP5 LSM confocal microscope with a 40× objective water lens and laser excitation at 635 nm; the filter set used was 700-740 nm.

### Mitochondrial Co-localization Assay

Co-localization of **PBB** and MitoTracker Green was studied in HepG2 cells using a confocal laser microscope. Briefly, 2 × 10^5^ HepG2 cells were seeded in 35 mm glass-bottom cell culture dishes. After incubation for 24 h, the cells were treated with **PBB** for 4 h. Then, the cells were washed with PBS and stained with MitoTracker Green for 30 min. The fluorescent images were recorded using the confocal microscope (Leica TCS SP5 LSM) with a 40× objective water lens; **PBB** laser excitation at 635 nm, filter set used was 480-530 nm; MitoTracker Green laser excitation at 561 nm and the filter set used was 580-630 nm.

### *In vitro* Anti-proliferation Assay

The* in vitro* anti-proliferative effects of **PBB** were tested on four human cancer cell lines (MCF-7, HeLa, HT29, and HepG2) and two normal cell lines (LO2 and CCD841) using the MTT assay. Briefly, **PBB** was dissolved in DMSO to obtain a 10 -mM stock solution, and diluted with cell culture medium to obtain different concentrations of **PBB** culture media (1.56, 3.125, 6.25, and 12.5 μM). The cells were seeded in 96-well plates with a density of 5 × 10^4^ cells/well and incubated for 24 h. Then, the old culture medium was replaced with a fresh **PBB** medium, and the cells were incubated for 48 h. After incubation, 10 µL of MTT solution (5 mg/mL MTT in PBS buffer) was added to each well, and the cells were incubated in the dark for another 4 h. Then, the medium was replaced with 200 μL DMSO to dissolve the formazan crystals. The absorbance was measured at 570 nm using a microplate reader.

### Intracellular ROS Detection

The level of intracellular ROS was detected using confocal imaging. HepG2 cells (1 × 10^4^/mL) were plated on a cell culture plate and incubated in an incubator containing 5% CO_2_ at 37 °C for 36 h, and were then incubated with **PBB** (15 μM) or PPT (15 μM) medium at 37 °C for another 4 h. 2,7-Dichlorodi -hydrofluorescein diacetate (DCFH-DA) (10 μM), a ROS capture agent, was added to the cells and incubated for 30 min. Finally, fluorescence images were captured using confocal microscopy, and the corresponding green fluorescence intensity of DCF was achieved; excitation at 488 nm and emission at 480-520 nm.

### Detection of Mitochondrial Membrane Potential

HepG2 cells (1 × 10^4^/mL) were seeded in cell culture plates and incubated in an incubator containing 5% CO_2_ at 37 °C for 24 h. The cells were then incubated with **PBB** (15 μM) or PPT (15 μM) medium at 37 °C for another 4 h. A solution of JC-1 (10 μg /mL in RPMI-1640 medium) was added to the cells, and they were incubated at 37 °C for 20 min. The cells were isolated and washed thrice with PBS. The cell images were recorded using a confocal fluorescence imaging system. For the green channel, the excitation wavelength was 488 nm and the emission wavelength was 510-545 nm. For the red channel, the excitation wavelength was 535 nm and the emission wavelength was 580-610 nm.

### Calcein-AM/ PI Staining

HepG2 cells (1.2 × 10^6^ cells/mL) were seeded in six-well plates and incubated in an incubator containing 5% CO_2_ overnight. Then, the cells were treated with a control solution (DMSO) and** PBB**, separately, for 6 h. Then, the cells (adherent and suspension cells) were collected, centrifuged at 2000 rpm for 5 min, and washed twice with cold PBS. Lastly, the cells were stained with Calcein-AM (green fluorescence, live-cell staining) and PI (red fluorescence, dead-cell staining). The fluorescence images were captured using the confocal fluorescence imaging system.

### *In vivo* Fluorescence Imaging and Surgery

HepG2 tumor-bearing mice were used for *in vivo* fluorescence imaging. When the tumor grew to ~300 mm^3^, **PBB** solution (20 mg/kg) was injected via the tail vein, after which the fluorescence intensity was observed using a Caliper IVIS Lumina II optical imaging system at different time points (1, 2, 4, 8, 12, 24 h, and 36 h). To further validate the intraoperative imaging feasibility, the subcutaneous tumor was removed using fluorescence for guidance.

### *Ex Vivo* Fluorescence Imaging and Quantification of Fluorescence in Major Organs

To further validate the biodistribution of **PBB**, the major organs (heart, lung, liver, kidney, spleen, colon, and tumor) were collected, and the fluorescence intensity was detected using the Caliper IVIS Lumina II optical imaging system.

### *In vivo* Tumor Growth Inhibition

HepG2 cells (1 × 10^6^) were subcutaneously injected in female BALB/c mice. When the tumor volumes reached 100 mm^3^, the tumor-bearing mice were randomly assigned to four groups - PBS (control), PTT (24 μmol/kg), and **PBB** (12 and 24 μmol/kg). The progression of the tumors and the body weight of the animals were monitored every 2 days until the 18^th^ day. Once the weight of the mice was reduced by 20% between observations, they were euthanized. The larger diameter (L) and smaller diameter (W) of the tumor were measured with a caliper, and tumor volume was calculated using the formula L × W^2^ × 0.5. At the end of the experiment, the mice were sacrificed, and the tumors were harvested and weighed. The tumors were fixed with 4% paraformaldehyde, followed by ethanol dehydration and paraffin embedding, and ultimately stained with H&E.

### *In vivo* Biosafety Assay

After treatment with **PBB** (9 mmol/kg), the mice blood was collected carefully and kept at 4 °C for 2 h. For the liver function tests, the blood was centrifuged (3000 rpm, 4 °C) for 10 min, and the blood serum was obtained to measure the activities of aspartate aminotransferase (AST) and alanine aminotransferase (ALT). The activities of AST and ALT were tested using commercial kits purchased from Nanjing Jiancheng Bioengineering Institute (Nanjing, China), and the assays were performed following the manufacturer's instructions. The liver tissue samples of the treated mice were subjected to 4% paraformaldehyde fixation, ethanol dehydration, paraffin embedding, and staining with H&E.

## Results and Discussions

### Synthesis of PBB

**Scheme 1:**
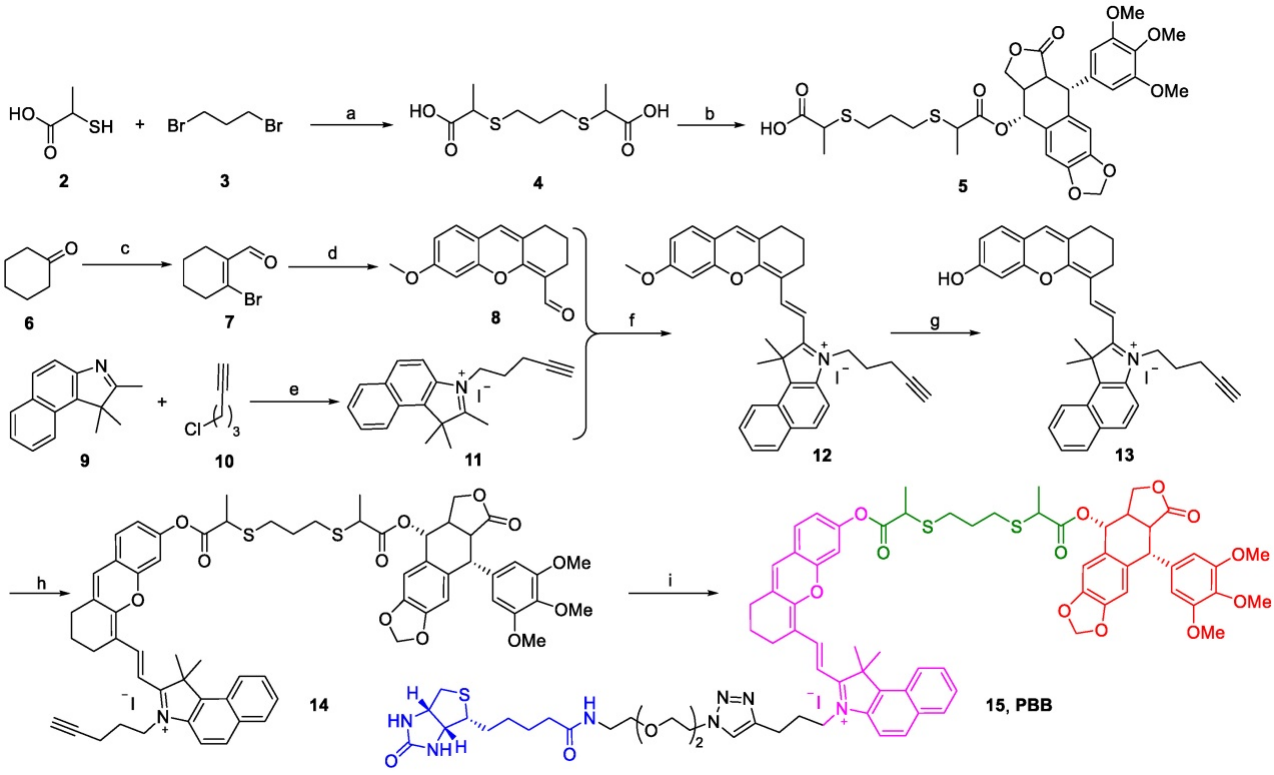
Synthetic scheme for **PBB**. (a) NaOH, MeOH, 0 ℃, 10 min, and then r.t. 12 h; (b) EDCI, DMAP, DCM, 4 h; (c) PBr_3_, DMF, CHCl_3_, 0 ℃, 30 min, and then rt 12 h; (d) 4-methoxy salicylaldehyde, Cs_2_CO_3_, DMF, 24 h; (e) KI, MeCN, reflux 24 h. (f) CH_3_COOH, Py, EtOH, rt 12 h; (g) BBr_3_, DCM, 12 h; (h) EDCI, DMAP, DCM, 4 h; (i) TBTA, Cu(CNCH_3_)_4_PF_6_, DCM, r.t., 10 h.

The chemical synthesis of PBB is summarized in Scheme 1. Firstly, 2-mercaptopropanoic acid (2) reacted with 1,3-dibromopropane (3) in the presence of NaOH in MeOH to yield linker (4), which was then treated with PPT under the condition of N-(3-Dimethylaminopropyl)-N'-ethylcarbodiimide hydrochloride (EDCI) and 4-dimethylaminopyridine (DMAP) to obtain the intermediate (5). Then, cyclohexanone (6) was treated with PBr3 in the solution of DMF and CHCl3 to afford compound (7). The xanthene skeleton was obtained according to a previous report 29, where the compound (7) was further treated with 4-methoxy salicylaldehyde to yield intermediate (8). Then, compound (11) was constructed by the compound (9) and (10) via quaternization. Compound (11) further reacted with the compound (8) in the presence of acetic acid and piperidine to obtain compound (12) via Knoevenagel reaction, and the compound (12) further underwent demethylation reaction with BBr3 to yield (13). The intermediate (14) was obtained by coupling (13) with (5) in the presence of EDCI and DMAP. Finally, intermediate (14) was treated with biotin-N3 in the presence of Cu(CNCH3)4PF6 via the "Click" reaction to obtain the final compound PBB (15), which was purified (purity >95%) and characterized using MS, 1H NMR, 13C NMR, and HRMS.

### GSH/ROS-Sensitivity of PBB and Drug Release

**Figure 2:**
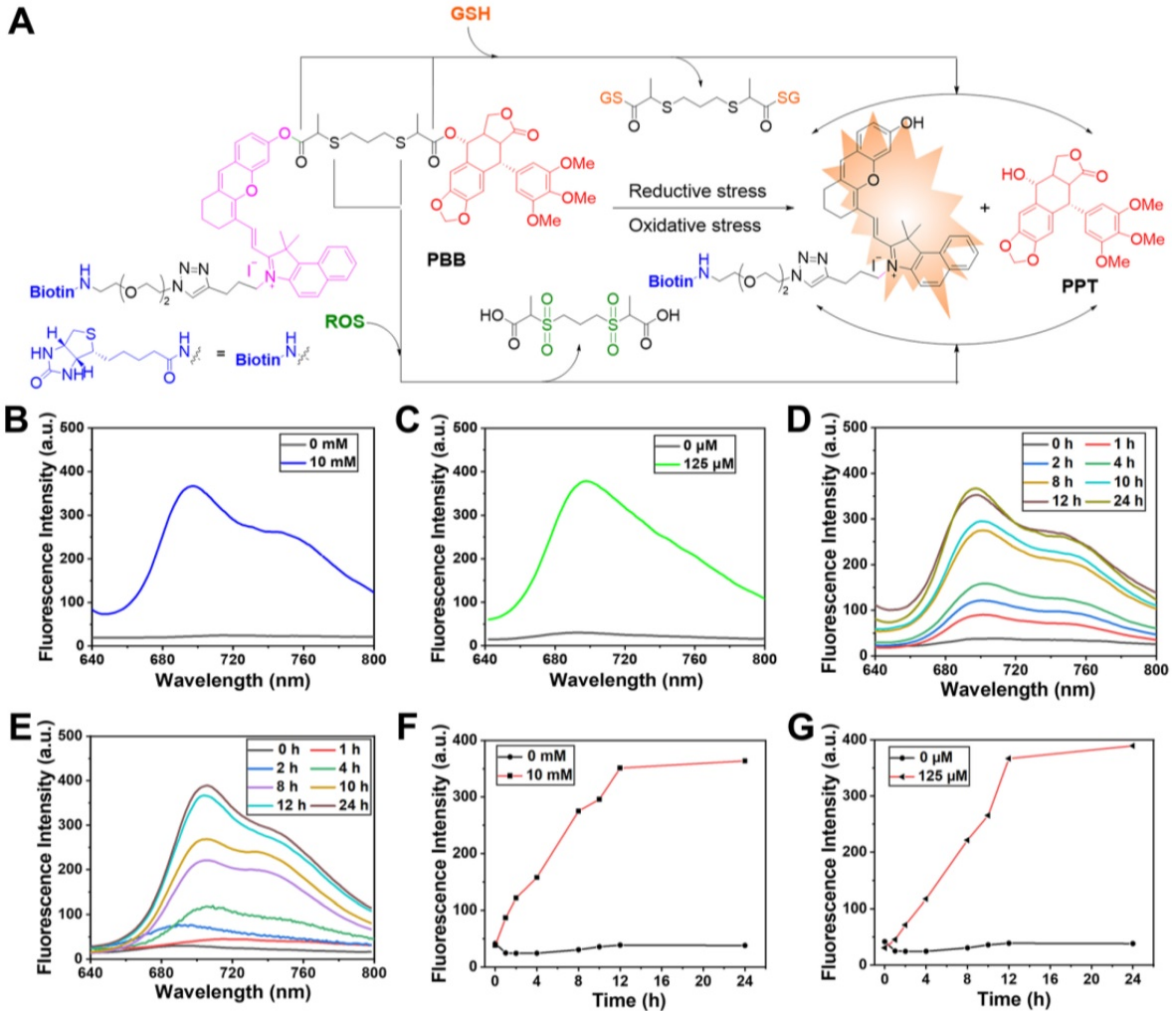
(A) Activation modes for **PBB** expected to be operative under reductive and oxidative stress conditions. (B) and (C) Fluorescence spectra of **PBB** (10 μM) exposed to 10 mM GSH and 125 μM ROS in deionized water (5% v/v DMSO). (D) and (E) Time-dependent changes in the fluorescence intensity upon incubation of **PBB** (10 μM) with GSH (10 mM) and H_2_O_2_ (125 μM) in deionized water (5% v/v DMSO). (F) and (G) Time -dependent changes in the fluorescence intensity (at 700 nm) of 10 μM** PBB** incubated with 10 mM GSH and 125 μM H_2_O_2_ in deionized water (5% v/v DMSO) (λ_ex_ = 635 nm).

The possible response mechanism of PBB to redox stress conditions was shown in Figure 2A. Thiolactate as a redox responsive subunit can be specifically cleaved by intracellular ROS or GSH. On the one hand, the thiolactate ester bond can be nucleophilically attacked by the sulfhydryl group of GSH, leading to the release of the active drugs, biotin-linked CyOH, and thiolactic GSH-substituted thioester. On the other hand, the oxidation of thiolactate linker by ROS leads to a sulfone moiety followed by hydrolysis, which, in turn, releases the active drug, biotin-linked CyOH, and oxided sulfonyl isopropyl acid 30. To study the NIR fluorescence and drug release behavior of PBB could sensitively occur under reductive and oxidative stress media (GSH and H_2_O_2_, respectively), the changes in UV absorption, fluorescence intensity, and the peak of high-performance liquid chromatography (HPLC) were monitored under the condition of GSH or H_2_O_2_ in phosphate-buffered saline (PBS) at 37 °C.

In the UV-*vis* spectrum, PBB exhibited the maximum absorbance at 635 nm. Upon the treatment with GSH, the absorption spectra of PBB demonstrated a increase at 635 nm (Figure S1A). Similarly, upon the treatment with ROS, the absorption of PBB increased sharply at 635nm (Figure S1B). In addition, the absorbance of PBB at 635 nm increased in a time-dependent manner (Figures S1C & S1D). In the fluorescence spectrum, incubation with 10 mM GSH or 0.125 mM H_2_O_2_ led to an apparent increase in the fluorescence intensity at 705 nm due to the GSH-triggered thiolysis or ROS-induced hydrolysis, both of which contributed to the targeted release of PPT and the fluorescent fragment (Figures 2B & 2C). These mimicked the activation conditions of the theranostic agent that were expected under physiological reductive stress. Besides, the fluorescence intensity at 700 nm was time-dependent (Figures 2F & 2G). For a given time, the fluorescence intensity increased in a concentration-dependent manner (Figures S2A & S2B).

**Figure 3:**
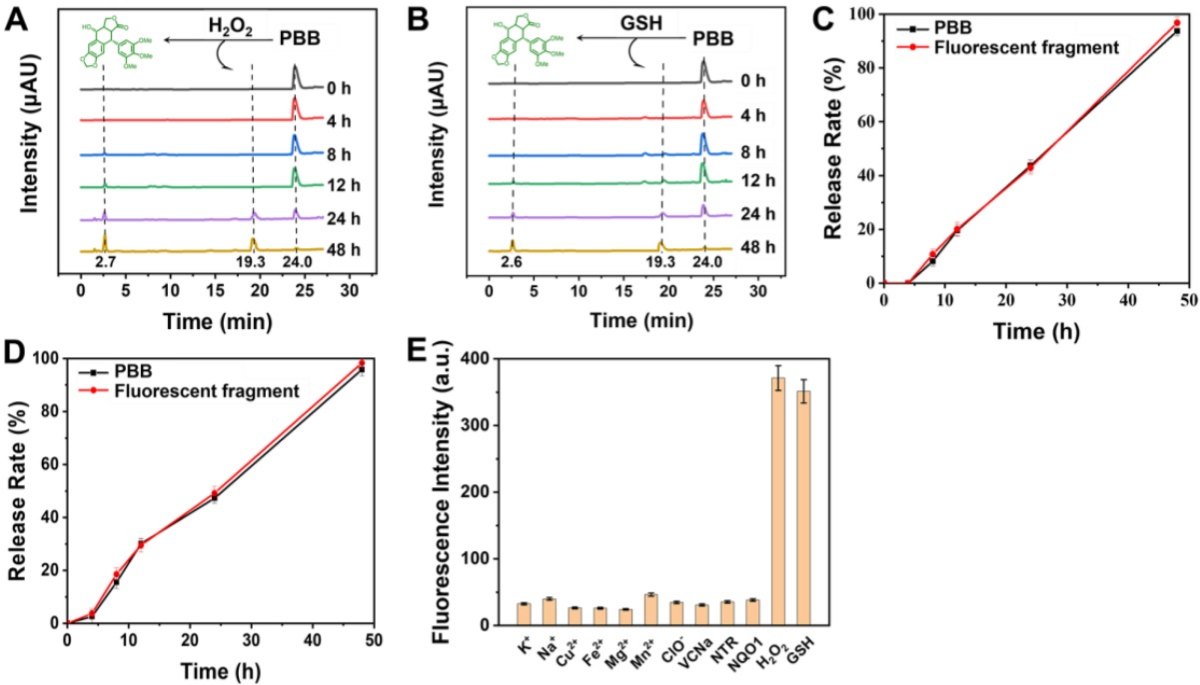
(A) and (B) HPLC of **PBB** (10 µM) and PPT were recorded at different time points (0, 4, 8, 12, 24, and 48 h ) after treatment with GSH (10 mM) and ROS (125 μM) separately in PBS at 37 °C. (C)* In vitro* release of GSH-responsive PPT and the fluorescent fragment (mean ± SD, n = 3). (D) *In vitro* release of H_2_O_2_-responsive PPT and the fluorescent fragment (mean ± SD, n = 3). (E) Fluorescence intensity of **PBB** (50 µM) treated with ROS, GSH, and various biological analytes (Na^+^, K^+^, Cu^2+^, Fe^2+^, Mg^2+^, VcNa, Mn^2+^, ClO^-^, nitroreductase, and NQO1) at 1 mM for 24 h in deionized water (5% v/v DMSO) (λ_ex_ = 635 nm) (mean ± SD, n = 3).

Furthermore, the release of active PPT and the fluorescent fragment was confirmed using HPLC. As depicted in Figures 3A & 3B, after incubation without GSH, a retention peak of **PBB** appeared at 24.0 min in the HPLC spectrum; however, as the retention time prolonged, two new peaks appeared at retention times 2.7 min (free PPT) and 19.3 min (hemicyanine fluorescent fragment). Incubation with GSH for 48 h led to the disappearance of the **PBB** peak. These results are illustrated in Figure 3, **PBB** underwent thiolysis of the phenolic ester moiety and released free PPT. Similarly, incubation of **PBB** with H_2_O_2_ resulted in the release of active PPT and the fluorescent fragment due to the oxidation of the thioether moiety to the sulfonyl group and further hydrolysis. ROS- and GSH- responsive fluorescent properties and active PPT release were suitable for selective visualizing and treating TME.

We then investigated whether the prodrug PBB was stable in human serum. Briefly, the samples were incubated at 37 °C and measured after 0, 0.5, 1, 2, 4, 8, 12, and 24 h using HPLC. The results showed that PBB was stable within 8 h when incubated at 37 °C in rat plasma, whereas the prodrug PBB underwent degradation after 8 h (Figure S2). Although PBB was unstable after 8 h, the eight hours of stability suggested that PBB could be used in elaborate biological experiments.

### Specific Response of PBB to GSH and ROS

Selective recognition is a very significant performance of specific activation. To verify the specific response of **PBB** toward GSH and ROS, a variety of physiologically relevant interferential species were analyzed. As shown in Figure 3E, after the addition of the inorganic salts (Na^+^, K^+^, Cu^2+^, Fe^2+^, Mg^2+^, and Mn^2+^), reductant (Vitamin C), oxidant (ClO^-^), and enzymes (nitroreductase and NQO1), the fluorescence intensity was found to be very low, while the fluorescence intensity increased significantly only after the addition of GSH or H_2_O_2_. These results suggested that **PBB** has a highly selective response to GSH and ROS. In other words, intracellular GSH and ROS enable **PBB** to release PPT without interference from other physiological molecular species.

### Cellular Selective Imaging of PBB

**Figure 4:**
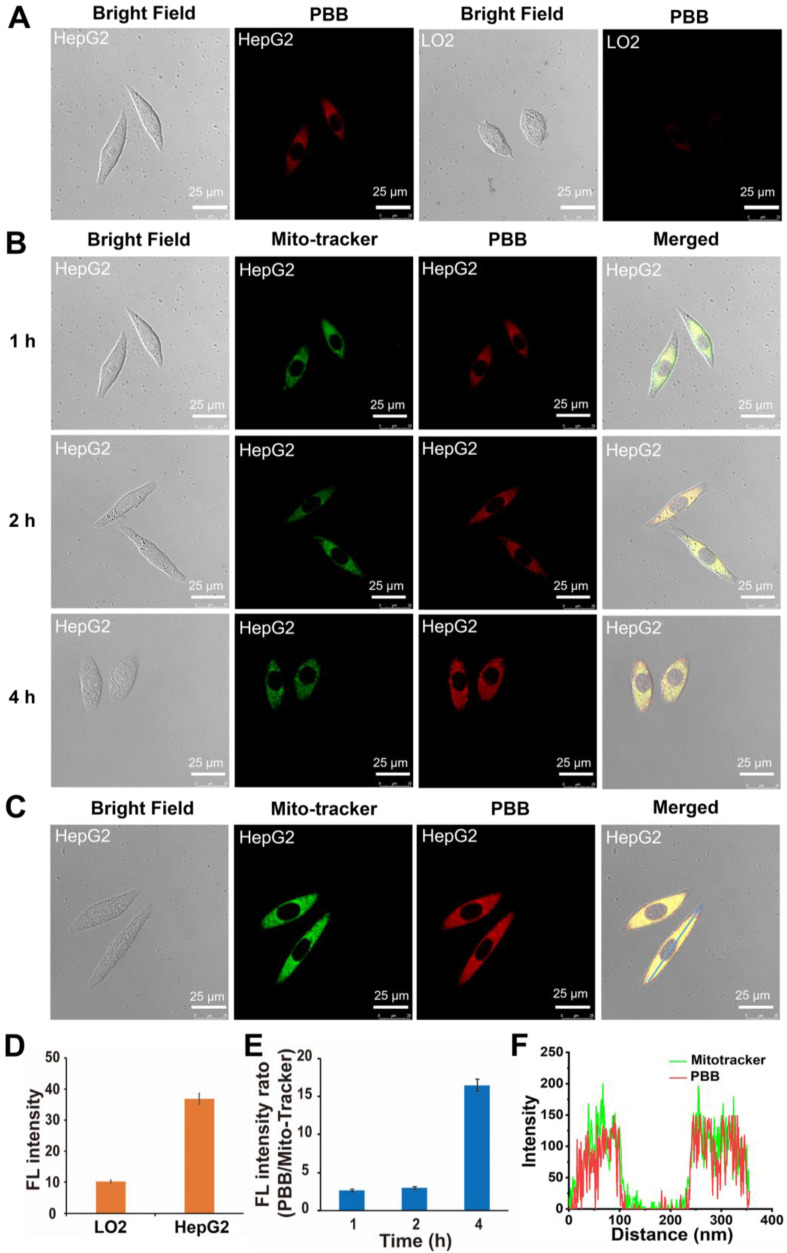
(A) Confocal microscopy images of **PBB** in human hepatoma (HepG2) cells and normal (LO2) cells. Confocal fluorescence imaging of **PBB** (10 μM) in HepG2 and LO2 cells after incubation for 4 h. Scale bars, 25 μm; λex, 635 nm; filter set, 700-740 nm. (B) The cellular uptake of **PBB** at different time points (1, 2, and 4 h). The cells were pre-treated with **PBB** (50 μM, red, λ_ex_ = 638 nm) for 30 min, and then incubated with MitoTracker Green (Green, λ_ex_ = 488 nm) for 1, 2, and 4 h. Scale bar for all images, 25 μm. (C) Fluorescence co-localization of **PBB** and MitoTracker Green. HepG2 cells were co-stained with** PBB** and Mito-Tracker Green. (D) The fluorescence intensity quantification of **PBB** in LO2 and HepG2 cells (mean ± SD, n = 3). (E) The fluorescence intensity quantification of **PBB** in HepG2 cells at different time points (mean ± SD, n = 3). (F) Co-localization of **PBB** (red) and Mitotracker Green (green).

Due to the overexpressed biotin receptors on the surface of cancer cells, biotin molecules and their conjugates are taken up favorably by the cancer cells 31. The tumor specificity of PBB was evaluated in human cancer HepG2 cells and normal LO2 cells. As shown in Figure 4A, a much stronger fluorescence was observed in HepG2 cells. The quantification of intracellular fluorescence showed that the fluorescence intensity in HepG2 cells was 6-fold higher than that in the LO2 cells, which supported the more tremendous amount of intracellular fluorescent fragment and PPT release because of the overexpressed GSH and/or ROS in the cancer cells. The results suggested that PBB -conjugated biotin selectively ignited hepatoma HepG2 cells rather than human normal cells LO2.

The cellular uptake efficiency was further determined by quantifying the intracellular fluorescence intensity of **PBB** in HepG2 cells at 1, 2, and 4 h post-treatment. The fluorescent intensity of MitoTracker Green was used as a reference standard. The fluorescence intensity ratio of **PBB** to MitoTracker Green was used as a quantifiable indicator. As expected, with the prolonging of incubating time, the intensity of red fluorescence in the HepG2 cells increased significantly (Figure 4B). The fluorescence intensity ratio increased with the incubation time (Figure 4E). These results suggested that the cellular uptake of **PBB** increased in a time-dependent manner.

### PBB Targeted the Mitochondria in Cancer Cells

Cationic fluorescent dyes, such as rhodamine 123 and tetramethylrhodamine, usually serve as mitochondrial trackers in living cells 32-35. Co-localization experiments were performed by co-staining the HepG2 cells with **PBB** and a commercially available mitochondrial probe MitoTracker Green. HepG2 cells were incubated with **PBB** (10 μM) and MitoTracker Green for 1 h to examine intracellular fluorescence activation. As shown in Figures 4C & 4F, the green fluorescence of MitoTracker Green overlapped with the red fluorescence of **PBB**, and Pearson's coefficient was 0.91 (Figure S3). These results indicated that **PBB** successfully targeted the mitochondria.

### *In vitro* Selective Anti-tumor Activities

**Figure 5:**
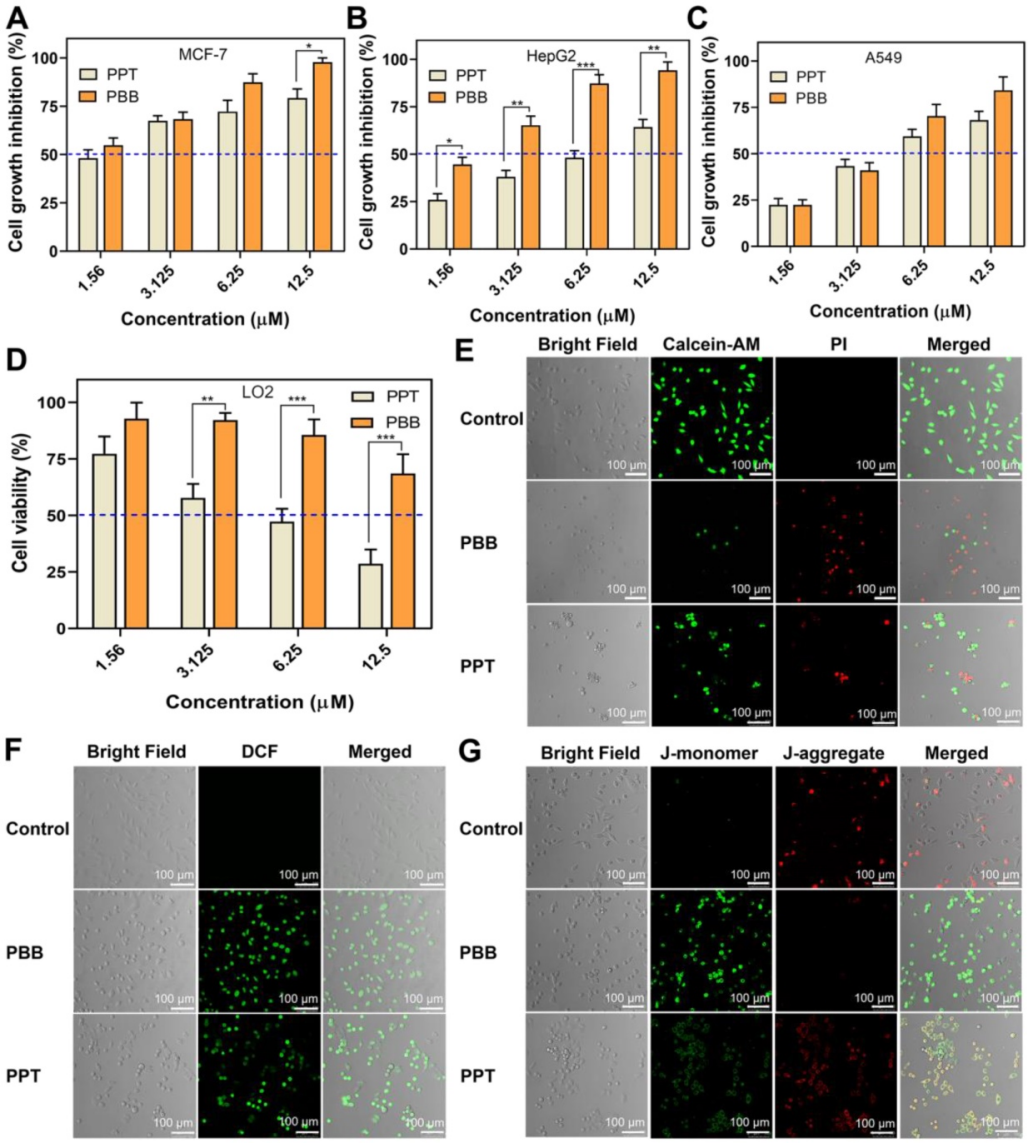
(A-D) Cytotoxicity of **PBB** in three cancer cell lines (MCF-7, A549, HepG2) and one normal cell line (LO2) (mean ± SD, n = 3; (mean ± SD, n = 3; ***p < 0.0002, **p < 0.0021, *p < 0.0332). (E) Fluorescence imaging of Calcein-AM- and PI -stained HepG2 cells after different treatments. (F) Fluorescence imaging of the probe DCFH-DA in HepG2 cells treated with PBS, PPT and **PBB**. (G) Fluorescence imaging of JC-1 in HepG2 cells.

As described above, PBB was ingested specifically by the cancer cells. **Accordingly,** the *in vitro* anti-proliferative efficacy of PBB was evaluated using three cancer cell lines (MCF-7, HepG2, and A549) and one normal cell line (LO2) by using the MTT assay (Figure 5A-5D). As anticipated, PBB and PPT inhibited the growth in the three cancer cell lines in a dose-dependent manner. PBB achieved more promising anti-tumor activity against three cancer cells than PPT at the same dose, especially significantly in MCF-7 cell line, with an inhibition rate above 50% at the 1.56 μM dose (Figure 5A). Furthermore, the cell growth inhibitory rates of PBB treated HepG2 cells at 1.56 and 3.125 μM doses are nearly twice more pronounced than PPT treated group (Figure 5B). In addition, compared to PPT, higher cell viabilities in PBB treated group were observed in the human normal cells LO2 as exemplified with cell viability above 50% at the high dose of 12.5 μM (Figure 5D). This result was reasonably attributed to the specificity of PBB to the HepG2 cells, resulting in the higher release of PPT and enhanced *in vitro* cytotoxicity in HepG2 cells. PBB is, therefore, an excellent targeting delivery agent due to the enhanced anti-proliferative activity in cancer cells and remarkably decreased toxicity in normal cells.

Moreover, Propidium iodide (PI) and Calcein-AM dual-staining, which can distinguish between the dead and living cells using red and green fluorescence, respectively, demonstrated that apoptosis occurred in **PBB** -treated cells (Figure 5E). Intracellular ROS accumulation and loss of mitochondrial transmembrane potential are typical characteristics of dead cells 36, 37. Figure 5F clearly showed that cells treated with **PBB** have higher fluorescence intensity, implying that **PBB** significantly induced intracellular accumulation of ROS. Furthermore, **PBB** induced MMP depolarization (Figure 5G). **T**hese observations were associated with cancer cell-specific cytotoxicity. These results revealed that **PBB** showed prominent and selective inhibitory activity in cancer cells, indicating that **PBB** exhibited good therapeutic efficacy *in vitro.*

### Visualizing Liver Tumors in Mouse Models via PBB

**Figure 6:**
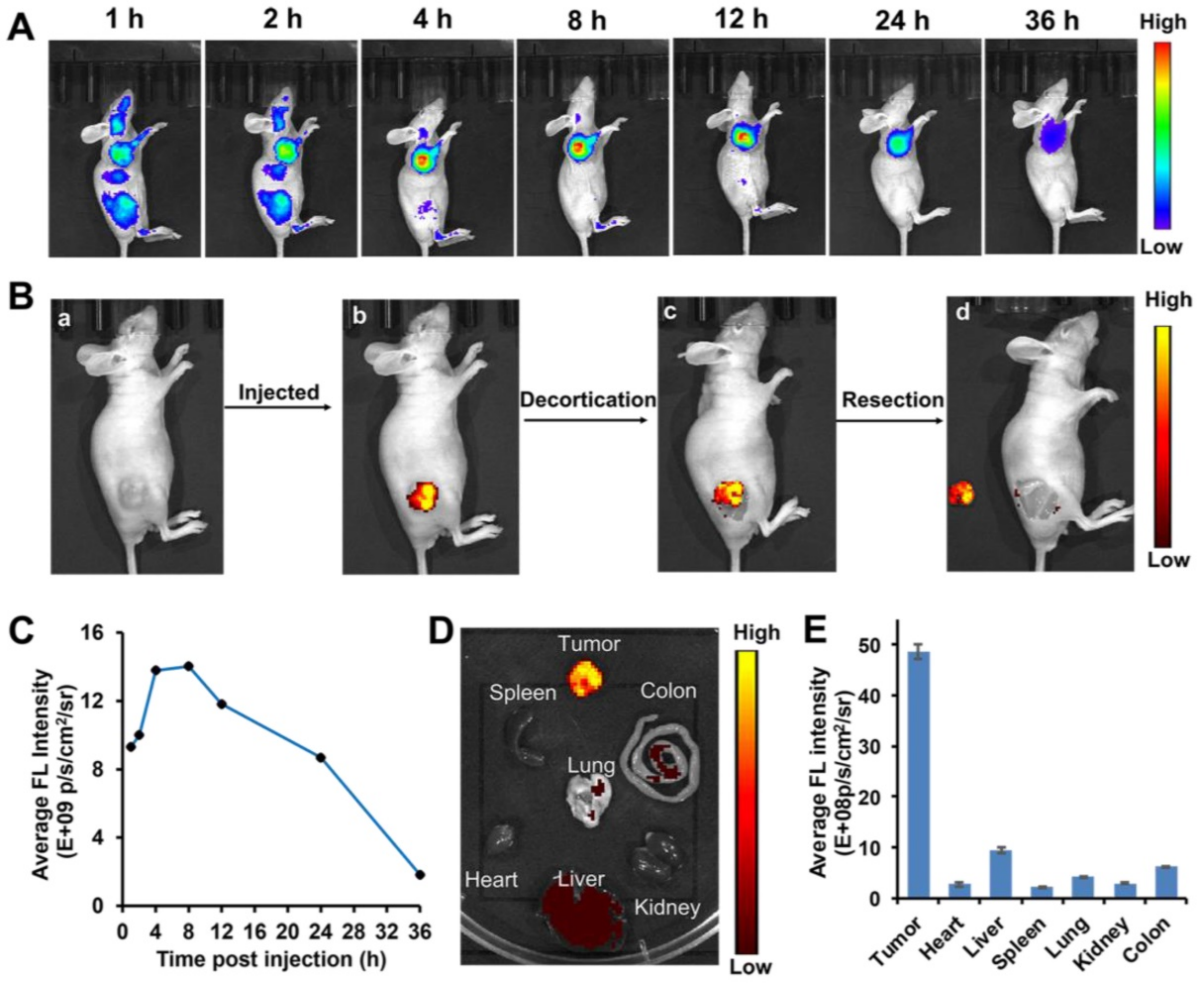
*In vivo* diagnostic efficacy of **PBB** in HepG2 tumor xenograft mice. (A) Fluorescence imaging of the tumor in the subcutaneous tumor mice model at various time points post-intravenous injection of **PBB** (20 mg/kg). (B) The procedure of NIR imaging-guided surgery. (C) The relative average fluorescence intensity after intravenous administration of **PBB** up to 24 h. (D) *Ex vivo* fluorescence imaging of the tumor and major organs. (E) The relative average fluorescence intensity in these tissues (tumor, heart, liver, spleen, lung, kidney, and colon) (mean ± SD, n = 3).

Considering its superior *in vitro* results in simultaneously targeting tumor cells and ROS/GSH-responsive activation, the targeting efficacy of **PBB** in nude mice xenograft model and the visualization of **PBB** in tumor tissues were investigated at different time intervals. As shown in Figure 6A, upon intravenous administration of **PBB** (20 mg/kg), high fluorescence intensity was observed in the liver and tumor at 1 and 2 h. After four hours of circulation in the nude mouse, the fluorescence signals mainly distributed in tumor position and reached to maximum at 8 h post injection. The tumor site displayed fluorescence signal continuously within 24 h, then was gradually faded. To further validate that **PBB** is specific to the tumor tissues, the fluorescent biodistribution of **PBB** was investigated using *ex-vivo* imaging. After 24 h of intravenous injection of **PBB** (20 mg/kg), the fluorescent intensity in the tumor tissue and major organs, including the heart, liver, lung, kidney, colon, and spleen, was examined. The fluorescent signal in the tumor was significantly greater than that in the other organs (Figure 6D). These results confirmed that **PBB** is capable of precisely targeting and tumor tissues in mice via non-invasive fluorescence imaging, and there was efficient retention of **PBB** in the tumor for over 24 h with promising selectivity and real-time visualization *in vivo*.

### Fluorescence-Guided Tumor Surgery of PBB in the Mice Model

After successfully locating and monitoring the tumor in the subcutaneous HepG2 tumor model through an optical imaging system, we performed NIR imaging-guided tumor surgery (Figure 6B). After 24 h following administration of **PBB**, the fluorescence of the tumor guided us to determine the location of the liver tumor. The tumor site displayed strong **PBB** fluorescence (Figures 6B (b and c)). We then excised the liver tumor navigated by NIR imaging, and the excised tumor is presented in Figure 6B (d). The results demonstrated that **PBB** fluorescence could successfully guide tumor surgery.

### Anti-tumor Effect of PBB *in vivo*

**Figure 7:**
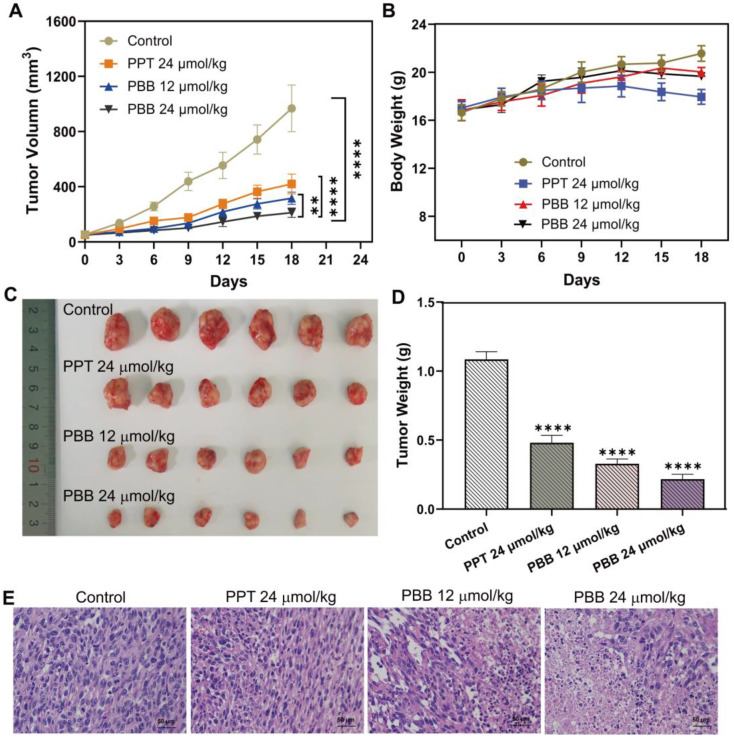
*In vivo* anticancer activity of **PBB** in HepG2 tumor xenograft nude mice. Mice were randomly assigned to one of the four groups - PBS (control), PPT (24 μmol/kg), **PBB** (12 μmol/kg), and **PBB** (24 μmol/kg). The respective treatments were intraperitoneally administrated every three days for 18 days. (A) Tumor volume growth curve over time in the four groups (mean ± SD, n = 6, **p < 0.0021, ****p < 0.0001). (B) Bodyweight growth curve over time in the four groups. (C) Excised tumors at day 18 after different treatments. (D) Average tumor weight on day 18 (mean ± SD, n = 6, ****p < 0.0001). (E) H&E staining of the tumors in the different groups.

The above cellular evaluation and *in vivo* imaging results demonstrated that **PBB** could precisely accumulate in tumor tissue and exert the tumor-selective and real-time NIR fluorescence imaging. Thus, the *in vivo* chemotherapeutic efficacy of **PBB** in HepG2 tumor xenograft nude mice was examined, and the changes in the tumor volume and weight were measured. Mice bearing the HepG2 tumor were randomly assigned to one of the four groups - PBS (control), PPT (24 μmol/kg), **PBB** (12 μmol/kg), and **PBB** (24 μmol/kg). The respective treatments were administered intraperitoneally every three days for 18 days. As shown in Figure 7A, the tumor volumes in the control group increased by more than 18-fold, while those in the **PBB** and PPT groups showed significantly inhibited tumor growth (Figure 7A). The tumor growth in the **PBB**-treated group was inhibited substantially. For example, the tumor growth inhibition (TGI) rate in the mice treated with a low dose of **PBB** (12 μmol/kg) was 67%, which was statistically more significant than that of PPT (24 μmol/kg). In the 24 μmol/kg **PBB** group, tumor growth was partially inhibited by 78%. The HepG2 tumor (Figure 7C) excised from the mice suggested that **PBB** shows anti-cancer activity. During the 18-day treatment, the weight of mice in the **PBB**-treated group showed negligible fluctuations, demonstrating almost no influence on the health of the mice. In addition, hematoxylin and eosin (H&E) staining of the tumor showed that **PBB** had a significant anti-proliferation effect on the tumor cells at an equivalent dose (24 μmol/kg). Therefore, in conclusion, **PBB** effectively inhibited tumor cell proliferation and induced apoptosis in the liver tumor tissue.

### Evaluation of the Safety Profiles of PBB *in vivo*

Balancing the toxicity and bioactivity of drugs is a major problem in clinical application. The clinical anti-cancer application of PPT is limited because of its high toxicity. Firstly, we evaluated the acute toxicity of PBB in mice. Acute toxicity of PBB was investigated along 24 hours after treating BALB/c mice with a single injection of different amounts (468.8, 421.9, 375.0, 337.5, 300 mg kg^-1^) of the PBB and different amounts (58.6, 46.9, 37.5, 30, 24 mg kg^-1^) of the PPT. As shown in Table S1, the median lethal dose (LD_50_) for PBB in mice was 376.7 mg/kg (with 95% confidence limits of 354.7-400.0), about 10.5-fold higher than PPT with the LD_50_ value of 35.9 mg/kg (with 95% confidence limits of 31.5-40.6). Consequently, the PBB was determined to be less toxic and have higher safety than PPT.

The hepatotoxicity of anti-tumor drugs is a common adverse effect. We further checked whether **PBB** effectively reduced the toxicity of PPT in the mouse tumor model. Liver function was evaluated using the indicators alanine aminotransferase (ALT) and aspartic transaminase (AST). H&E staining of the liver tissue was performed to evaluate the histopathological features. As depicted in Figures 7A & 7B, after a single intraperitoneal dose (9 mmol/kg) of PBS or **PBB**, no morphological or histopathological damage to the liver was observed in the PBS- and **PBB**-treated groups, while the mice treated with PPT presented significant liver necrosis, indicating that **PBB**, at 9 mmol/kg, was not toxic to the liver. Furthermore, liver function analysis showed no evident increase in the levels of AST and ALT in the **PBB**-treated mice. In contrast, both AST and ALT levels were elevated in the PPT-treated group, indicating that** PBB** showed no obvious nephrotoxicity in the tested mice compared to the PPT-treated group. These results showed that **PBB** reduced the toxicity of PPT.

## Conclusions

In summary, a smart targeting theranostic agent **PBB** integrating intraoperative near-infrared fluorescence diagnostic and chemotherapeutic functions was developed for cancer diagnosis and therapy. The accurate detection of tumors is essential in tumor surgery. Our study indicated that well-designed** PBB** not only displayed dual ROS/GSH -responsive abilities but also selectively inhibited the proliferation of the tumor. **PBB** accurately located the tumor *in vivo*, selectively and precisely illuminated tumor tissue, and could be used as an imaging-guided tool for surgical resection of the tumor tissue in mice. Furthermore, **PBB** simultaneously provided more effective chemotherapy than PPT by inhibiting hepatic tumor growth. In addition, **PBB** minimized the adverse effects of PPT and showed no sign of toxicity on the liver tissue. These results suggested that **PBB** integrated highly selective tumor diagnosis and efficient chemotherapy may be a useful tool for clinical accurate cancer therapy.

## Supplementary Material

Supplementary methods and figures.

## Figures and Tables

**Figure 1:**
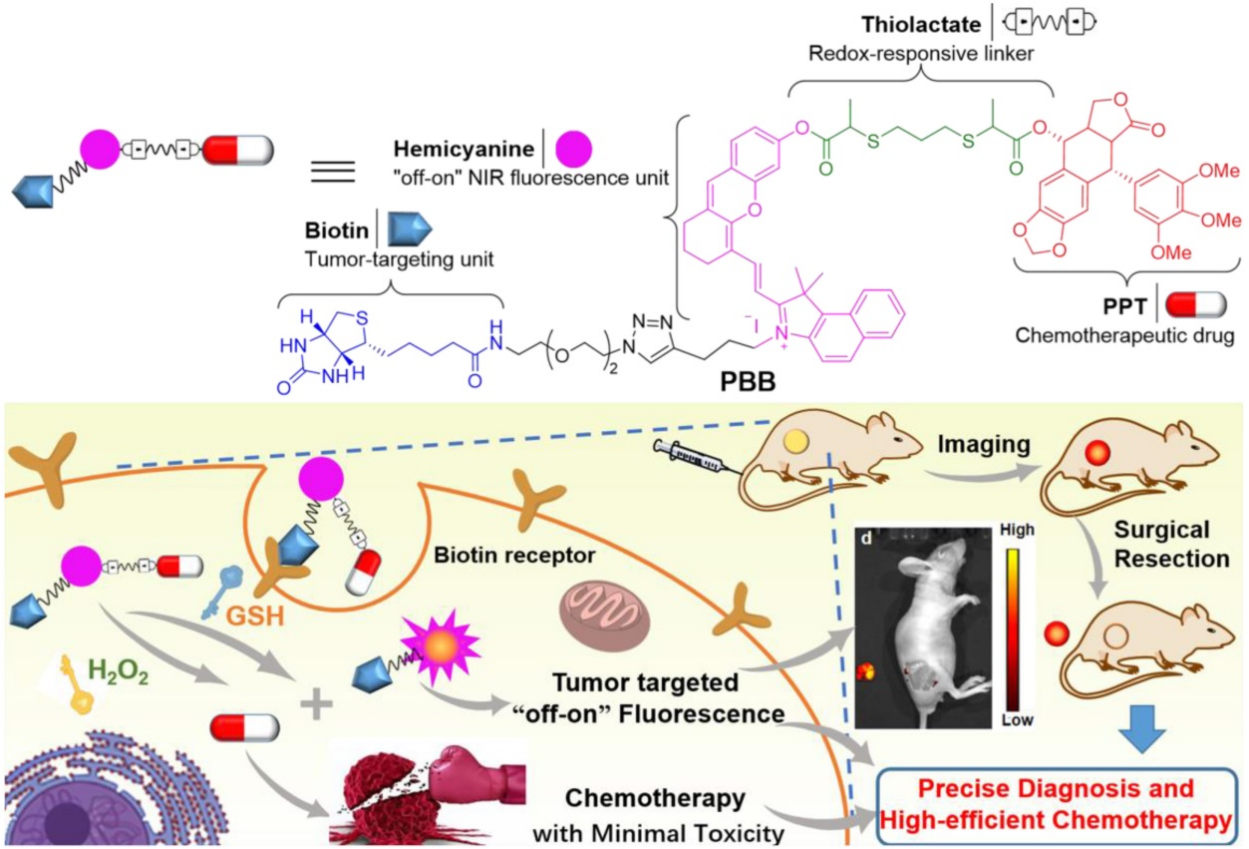
Chemical structure of TME-targeting and ROS/GSH-activated theranostic agent **PBB,** and its representative working principle in cancer cells.

**Figure 8:**
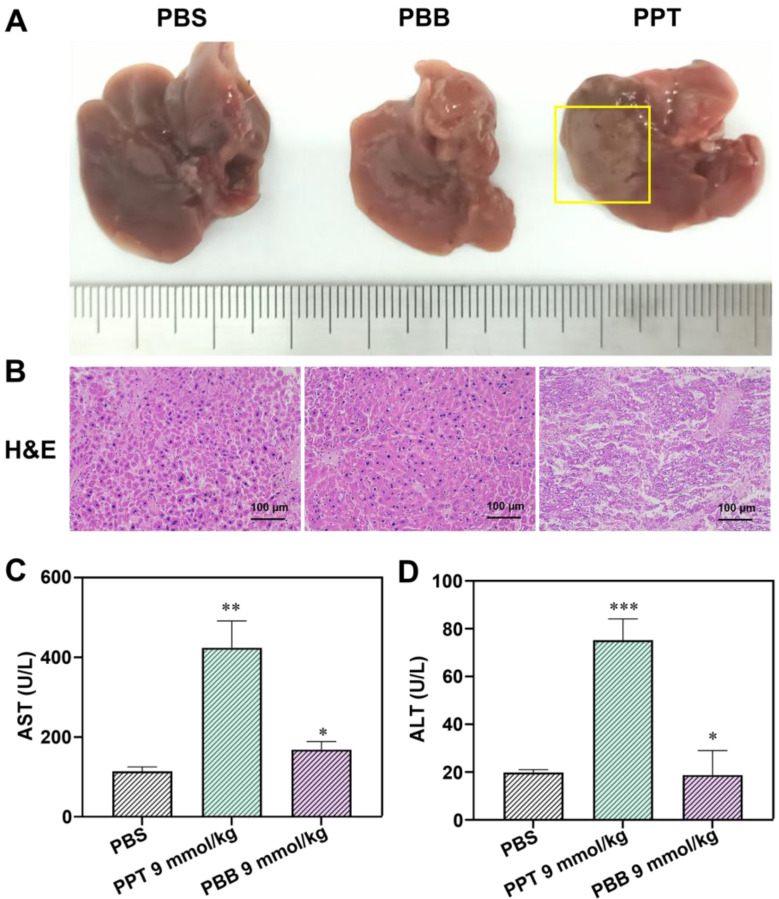
(A) Representative liver tissues from each treatment group. (B) H&E -stained images of the liver histology. (C) The levels of AST in the three treatment groups (mean ± SD, n = 3, **p < 0.0021, *p < 0.0332). (D) The levels of ALT in the three treatment groups (mean ± SD, n = 3, ***p < 0.0002, *p < 0.0332).

## Abbreviations

AST: aspartate aminotransferase
ALT: alanine aminotransferase
CyOH: hemicyanine
DMEM: dulbecco's modified eagle medium
DCFH-DA: 2,7-Dichlorodi-hydrofluorescein diacetate
DMAP: 4-dimethylaminopyridine
EDCI: N-(3-Dimethylaminopropyl)-N'-ethylcarbodiimide hydrochloride
GSH: glutathione
HPLC: high performance liquid chromatography
H&E: hematoxylin and eosin
HRMS: high resolution mass spectrum
JC-1: 5,5′,6,6′-Tetrachloro-1,1′,3,3′-tetraethyl-imidacarbocyanine
LD_50_: median lethal dose
MMP: mitochondrial membrane potential
MARCA: Model Animal Research Center Affiliated
MTT: methyl thiazolyl tetrazolium
NIR: near-infrared
NQO1: NAD(P)H: quinone oxidoreductase 1
PPT: podophyllotoxin
ROS: reactive oxygen species
TGI: tumor growth inhibition
TME: tumor microenvironment
UV: ultraviolet
