# Supplementary material for: TME-targeting theranostic agent uses NIR tracking for tumor diagnosis and surgical resection and acts as chemotherapeutic showing enhanced efficiency and minimal toxicity
Source: Theranostics. 2022 Feb 28;12(6):2535–48. doi: 10.7150/thno.68074 (PMC8965474; doi:10.7150/thno.68074)
Supplement: Supplementary file 1 — Supplementary methods and figures. [file thnov12p2535s1.pdf]

Supporting information

TME-targeting theranostic agent uses NIR tracking for tumor diagnosis and surgical resection and acts as chemotherapeutic showing enhanced efficiency and minimal toxicity

Zhongyuan Xu [1,#], Jianqiang Qian [1,#], Chi Meng [1,#], Yun Liu [1], Qian Ding [1], Hongmei Wu [1], Peng Li [2], Fansheng Ran [1], Gong-Qing Liu [1], Yunyun Wang [1,*], Yong Ling [1,2,*]

1. School of Pharmacy and Jiangsu Province Key Laboratory for Inflammation and Molecular Drug Target, Nantong University, Nantong 226001, China.
2. The Affiliated Hospital of Nantong University, Nantong University, Nantong 226001, People's Republic of China.

Supplemental Methods

Synthesis and characterization

All chemical reagents were purchased from commercial sources and used without further purification. All reactions were carried out under an air atmosphere unless stated otherwise. The purity of compounds was analyzed using HPLC (Waters 1525 binary HPLC pump with a Waters 2998 photodiode array detector) on an XBridge-C18 analytic column (5 μm, 4.6 mm × 150 mm). Mass spectra were recorded on a Mariner mass spectrometer (ESI).The ^{1}H and ^{13}C NMR spectra were obtained on a Bruker AV 400 M spectrometer, TMS as the internal standard. HRMS data of tested compounds were obtained on a JMS-SX102A (FAB) or LC/MSD TOF. UV−*vis* spectra were obtained on a spectrometer (UV1800PC, Jinghua, China).

Stability of ***PBB*** *in rat plasma*

Rat plasma was prepared before the experiment and maintained at -80 °C [1]. **PBB** (100 μg/ml) standard solution was added to 30% rat plasma in PBS (0.5% DMSO), vortex and shake to mix. Then the mixture was placed at 37 ℃ constant temperature for appropriate times (0 h, 0.25 h, 0.5 h, 1.0 h, 2.0 h, 4.0 h, 8.0 h, 12.0 h and 24.0 h) in the shaking box. After vortexing at 0 ℃ for 2 min, the centrifuge was handled at 12000 rpm/min for 5 min to gather the supernatant. The metabolic stability of PBB in rat plasma was measured and recorded by HPLC.

Synthetic Procedures

Compound 4

1,4-Dibromopropane (4.04 g, 20 mmol) was added to the pre-prepared 40 ml methanol with NaOH (3.48 g, 84.2 mmol) and thiolactic acid (4.37g, 41.2 mmol). The mixture was stirred for 12 h. Then the pH value of the mixture was adjusted to 4 using hydrochloric acid (2 mol/L). The mixture was extracted with DCM (300 ml) and washed with brine. The organic layer was collected and concentrated under reduced pressure to obtain colorless oil. The residue was purified on silica gel column eluted EtOAc/hexanes (1:1) to give the desired product **4** (colorless oil, 4.9 g, 97.2%). ^1H NMR (400 MHz, $CDCl_3$) δ 10.71 (s, 2H, 2COOH), 3.43 (q, *J* = 7.1 Hz, 2H, CH_2), 2.90 (m, 4H, $2CH_2$), 2.01 (m, 2H, CH_2), 1.47 (d, *J* = 7.2 Hz, 6H, $2CH_3$).

Compound 5

Compound **4** (200 mg, 0.48 mmol) was dissolved in 4 mL anhydrous DCM, and then EDCI (183 mg,0.96 mmol), PPT (362.9 mg,1.44 mmol) and DMAP (36 mg, 0.24 mmol) were added to the compound **4**-DCM solution, successively. The mixture was stirred for 12 h. After reaction, the mixture was extracted with 60 mL DMC and washed with brine for three times. The organic layer was collected and the solvent was removed by reduced pressure distillation. The residue was purified by columm chromatography with EtOAc/hexanes (1:5) on silica gel, the pure white solid (compound **5**) was afford (243 mg, 78 %). ^1H NMR (400 MHz, $CDCl_3$) δ 6.84 (d, J = 6.3 Hz, 1H, ArH), 6.55 (s, 1H, ArH), 6.39 (m, 2H, 2ArH), 6.00 (dd, J = 6.3, 1.3 Hz, 2H, CH_2), 5.91 (d, J = 8.8 Hz, 1H, CH), 4.61 (d, J = 4.7 Hz, 1H, CH), 4.39 (t, J = 7.4 Hz, 1H, CH), 4.22 (t, J = 9.6 Hz, 1H, CH), 3.81 (s, 3H, OCH_3), 3.76 (d, J = 3.5 Hz, 6H, $2OCH_3$), 3.52 (m, 1H, CH), 3.39 (m, J = 9.2, 6.8, 2.8 Hz, 1H, CH), 3.01 (m, 2H, CH_2), 2.82 (m, 4H, $2CH_2$), 1.99 (m, 2H, CH_2), 1.50 (m, 6H, $2CH_3$).

Compound 7

PBr_3 (5 mL) was added to a mixed solution of DMF (4.48 mL) in $CHCl_3$ (20 mL) which was pre-cooled at 0 ℃ and the mixture was stirred for 30 min, cyclohexanone (4.9 mL) was then added. The reaction was stirred at room temperature overnight. The mixture was poured into saturated K_2CO_3 solution and extracted with DCM for three times. The organic layer was dried over sodium sulfate and evaporated under reduced pressure to obtain compound **7** as a pale yellow solid pale yellow oil (4.9 g, 97.2 %).

Compound 8

Compound **7** (0.204 g, 1.08 mmol) was dissolved in 6 mL DCM, and 4-methoxysalicylic aldehyde (0.137 g, 0.9 mmol) and $CsCO_3$ (0.88 g, 2.7 mmol) were added to the DCM solution, which was stirred at room temperature for 24 h. Then the mixture was poured into brine and extracted with DCM (100 mL) for three times. The organic layer was dried over sodium sulfate and evaporated under reduced pressure. The residue was purified by column chromatography with EtOAc/hexanes (1:10) on silica gel to afford the product **8** as a pale yellow solid (0.847 g, 66.7 %).

Compound **11**

Compound **9** (500 mg, 3.14 mmol), KI (1.2g, 3.14 mmol) and chloropentyne (648.4 mg, 6.28 mmol) was dissolved in 5 mL MeCN, and the mixture was refluxed at 80 ℃ for 12 h. After reaction, the mixture was poured into water and extracted with DCM (100 mL) for three times. The organic layer was combined and dried over sodium sulfate and evaporated under reduced pressure. The residue was purified by column chromatography with EtOAc/hexanes (1:5) on silica gel to afford the product **11** as a gray black solid (1.03 g, 81.5 %).

Compound **12**

Compound **8** (610.2 mg, 2.52 mmol) was added to a solution of **11** (1.01 g, 2.52 mmol) in 5 mL methanol, 2 drops of piperidine was then added and the mixture was refluxed at 80 ℃ for 12 h. The solution was evaporated under reduced pressure. The residue was purified by column chromatography with DCM/MeOH (25:1) on silica gel to afford the blue product **12** (0.84 g, 79.1 %). ^{1}H NMR (400 MHz, $CDCl_3$) δ 8.76 (d, *J* = 14.9 Hz, 1H, ArH), 8.26 (d, *J* = 8.4 Hz, 1H, ArH), 8.05 (d, *J* = 8.9 Hz, 1H, ArH), 8.00 (d, *J* = 8.1 Hz, 1H, ArH), 7.83 (d, *J* = 8.9 Hz, 1H, ArH), 7.70 (m, 1H, ArH), 7.60(m, 1H, CH=C), 7.39 (d, *J* = 8.3 Hz, 1H, ArH), 7.24 (s, 1H, CH=C), 6.91 (dt, *J* = 8.3, 2.4 Hz, 2H, ArH), 6.69 (d, *J* = 14.9 Hz, 1H, CH=C), 4.80 (m, 2H, CH_2), 4.11 (m, 1H, C≡CH), 4.01 (m, 3H, OCH_3), 2.86 (m, 2H, CH_2), 2.79(m, 2H, CH_2), 2.57 (m, 2H, CH_2), 2.25(m, 2H, CH_2), 2.13 (m, 2H, CH_2), 2.12 (d, *J* = 4.8 Hz, 6H, $2OCH_3$), 1.99 (m, 2H, CH_2).

Compound **13**

To a stirred solution of **12** (300 mg, 0.73 mmol) in 5 mL DCM, 5 mL BBr_3 was slowly added under a nitrogen atmosphere at 0 ℃. The reaction was then stirred at room temperature for 24 h. After completion, the mixture was poured into ice water and adjusted the pH value to neutral with solid $NaHCO_3$. Then the mixture was extracted with DCM (60 mL) and washed with water for three times. The organic layer was separated, dried over anhydrous sodium sulfate, filtered and evaporated under reduced pressure. The residue was purified by column chromatography with DCM/MeOH (20:1) on silica gel to afford the blue product **13** (210 mg, 73 %). ^{1}H NMR (400 MHz, $CDCl_3$) δ 8.76 (d, *J* = 14.9 Hz, 1H, ArH), 8.26 (d, *J* = 8.4 Hz, 1H, ArH), 8.05 (d, *J* = 8.9 Hz, 1H, ArH), 8.00 (d, *J* = 8.1 Hz, 1H, ArH), 7.83 (d, *J* = 8.9 Hz, 1H, ArH), 7.70 (m, 1H, ArH), 7.60(m, 1H, CH=C), 7.39 (d, *J* = 8.3 Hz, 1H, ArH), 7.24 (s, 1H, CH=C), 6.91 (dt, *J* = 8.3, 2.4 Hz, 2H, ArH), 6.69 (d, *J* = 14.9 Hz, 1H, CH=C), 4.80 (m, 2H, CH_2), 4.11 (m, 1H, C≡CH), 4.01 (m, 3H, OCH_3), 2.86 (m, 2H, CH_2), 2.79(m, 2H, CH_2), 2.57 (m, 2H, CH_2), 2.25(m, 2H, CH_2), 2.13 (m, 2H, CH_2), 2.12 (d, *J* = 4.8 Hz, 6H, $2OCH_3$), 1.99 (m, 2H, CH_2).

Compound **14**

To a solution of **5** (243 mg, 0.37 mmol) in 4 mL anhydrous DCM, EDCI (141.3 mg, 0.74 mmol), **13** (204 mg, 0.34 mmol) and DMAP (28.5 mg, 0.19 mmol) was added

and the mixture was stirred at room temperature for 12 h. Then the mixture was quenched with saturated brine and extracted with 60 mL DCM. The residue was purified by column chromatography with DCM/MeOH (20:1) on silica gel to afford the blue product **14** (210 mg, 73 %). ^{1}H NMR (400 MHz, $CDCl_3$) δ 8.66 (d, *J* = 23.5 Hz, 1H, ArH), 8.19 (m, 1H, ArH), 7.99 (s, 1H, ArH), 7.94 (d, *J* = 8.2 Hz, 1H, ArH), 7.86 (m, 1H, ArH), 7.62 (m, 1H, ArH), 7.51 (m, 1H, ArH), 7.31 (m, 1H, ArH), 7.10 (m, 1H, ArH), 6.99 (d, *J* = 22.0 Hz, 2H, 2ArH), 6.86 (d, *J* = 15.3 Hz, 1H, ArH), 6.74 (d, *J* = 4.2 Hz, 1H, ArH), 6.46 (s, 1H, CH), 6.31 (s, 2H, 2CH), 5.91 (d, *J* = 6.2 Hz, 2H, CH_2), 5.81 (d, *J* = 8.1 Hz, 1H, CH), 4.91 (t, *J* = 7.1 Hz, 1H, CH), 4.53 (s, 1H, CH), 4.36 (m, 1H, CH), 4.18 (m, 1H, CH), 3.73 (s, 3H, OCH_3), 3.71 (m, 6H, $2OCH_3$), 3.57 (d, *J* = 7.5 Hz, 1H, CH), 3.39 (d, *J* = 7.3 Hz, 2H, CH_2), 2.82 (dd, *J* = 11.9, 5.4 Hz, 5H, CH, $2CH_2$), 2.76 (m, 2H, CH_2), 2.66 (d, *J* = 3.6 Hz, 4H, $2CH_2$), 2.16 (d, *J* = 7.0 Hz, 2H, CH_2), 2.01 (d, *J* = 3.8 Hz, 6H, $2CH_3$), 1.96 (m, 6H, $2CH_3$), 1.53 (d, *J* = 6.9 Hz, 2H, CH_2), 1.42 (s, 2H, CH_2).

*Compound **15***

To a solution of **14** (200 mg, 0.16mmol) in DCM, TBTA (22.3 mg, 0.054 mmol) and $Cu(CNCH_3)_4PF_6$ (2.7 mg, 0.0108 mmol) were added and stirred under a nitrogen atmosphere for 10 min, then Biotin-PEG-N_3 (58 mg, 0.132 mmol) was added. The resulting mixture was stirred at room temperature for 24 h. Then the mixture was poured into water and extracted with DCM (10 mL) for three times. The organic layer was dried over sodium sulfate and evaporated under reduced pressure. The residue was purified by column chromatography with DCM/MeOH (50:1) on silica gel to afford the final product **18** as a blue powder (228.8mg, 87 %). ^{1}H NMR (400 MHz, $CDCl_3$) δ 8.75 (d, *J* = 15.1 Hz, 1H, ArH), 8.27 (d, *J* = 8.0 Hz, 1H, ArH), 8.01 (dd, *J* = 15.9, 8.2 Hz, 2H, 2ArH), 7.75 (m, 2H, 2ArH), 7.63 (m, 2H, 2ArH), 7.34 (d, *J* = 8.5 Hz, 2H, ArH, CH), 7.02 (t, *J* = 7.9 Hz, 2H, 2ArH), 6.82 (d, *J* = 5.2 Hz, 1H, CH), 6.56 (dd, *J* = 18.7, 6.7 Hz, 2H, 2ArH), 6.39 (d, *J* = 1.5 Hz, 3H, CH, 2NH), 5.99 (t, *J* = 6.2 Hz, 3H, CH, CH_2), 5.89 (d, *J* = 8.6 Hz, 2H, CH_2), 4.60 (d, *J* = 3.6 Hz, 2H, CH_2), 4.46 (m, 2H, CH_2), 4.18 (dd, *J* = 18.7, 8.5 Hz, 2H, CH_2), 3.88 (m, 15H, $3OCH_3$, $3CH_2$), 3.49 (dd, *J* = 9.6, 5.8 Hz, 1H, CH), 2.99 (m, 18H, $9CH_2$), 2.37 (m, 3H, CH_2, CH), 2.17 (d, *J* = 7.5 Hz, 1H, CH), 2.13 (m, 13H, $5CH_2$, CH_3), 1.61 (dd, *J* = 13.3, 6.5 Hz, 4H, $2CH_2$), 1.52 (t, *J* = 8.2 Hz, 5H, CH_2, CH_3), 1.46 (m, 3H, CH_3), 0.92 (m, 5H, CH_2, CH_3); ^{13}C NMR (101 MHz, $CDCl_3$) δ 173.6, 173.4, 173.3, 172.4, 171.7, 153.1, 152.7, 148.0, 147.7, 146.7, 138.407, 137.1, 134.9, 132.3, 130.2, 128.1, 127.7, 126.7, 124.1, 119.8, 116.3, 113.2, 109.7, 108.0, 106.9, 105.9, 105.4, 105.1, 101.7, 77.4, 77.1, 76.8, 74.3, 71.3, 68.2, 67.8, 60.8, 56.1, 52.8, 48.8, 45.5, 43.7, 41.6, 41.3, 41.1, 41.1, 38.6, 38.5, 31.9, 30.4, 30.3, 29.7, 29.4, 28.7, 28.6, 27.9, 27.2, 24.1, 22.7, 20.2, 17.1, 17.1, 17.0, 14.2. HRMS (ESI): *m/z* calcd for $C_{83}H_{99}N_7O_{17}S_3^{+}$: 1561.6260; found: 1561.6244 $[M]^{+}$.

Table S1 . The oral acute toxicity of **PBB** and PPT in mice

Group	Dose (mg/kg)	Log dose	Total number	Mortality (%)	LD50 (mg/kg)	95% confidence limits
**PBB**	468.8	2.67	10	100	376.7	354.7—400.0
	421.9	2.63	10	70		
	375.0	2.57	10	60		
	337.5	2.48	10	20		
	300.0	2.38	10	0		
**PPT**	58.6	1.77	10	100	35.9	31.5—40.6
	46.9	1.67	10	70		
	37.5	1.57	10	50		
	30.0	1.48	10	40		
	24.0	1.38	10	0		

Figures S1-S11

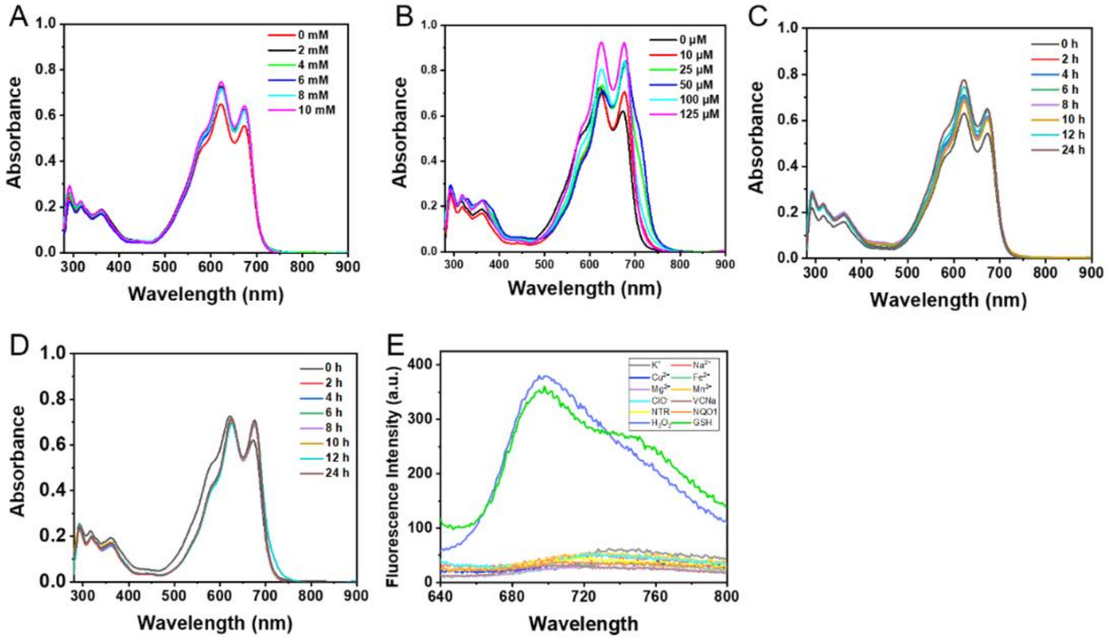

Figure S1. UV-*vis* absorbance spectra of **PBB** (10 μM) was recorded upon treatment with 5 mM GSH (A) and 125 μM H_2O_2 (B) in PBS (5% DMSO v/v) at 37 °C. UV-*vis* absorbance spectra of **PBB** was recorded upon treatment with GSH (C) and H_2O_2 (D) at different times (0, 2, 4, 6, 8, 10, 12, 24 h). (E) Fluorescence intensity of **PBB** was recorded upon treatment with ROS, GSH and various biological analytes.

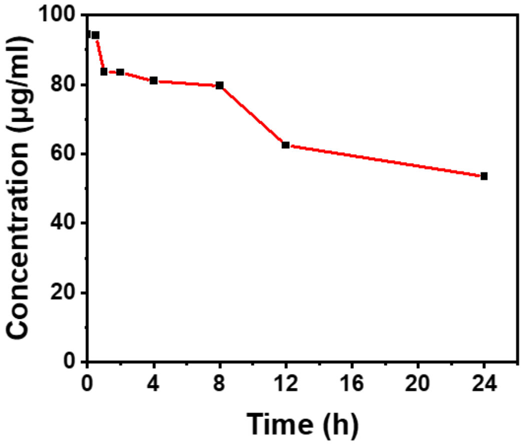

Figure S2. Stability measurements of **PBB** incubated in rat plasma at 37 °C and analyzed by HPLC at different times. The y-axis shows the relative concentration of the integrated peak areas of **PBB**.

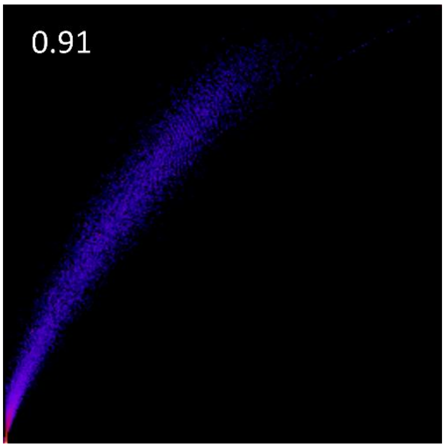

Figure S3. Scatter plot showing the overlap coefficient between **PBB** and Mitortracker Green (illustration: calculated Pearson correlation coefficient).

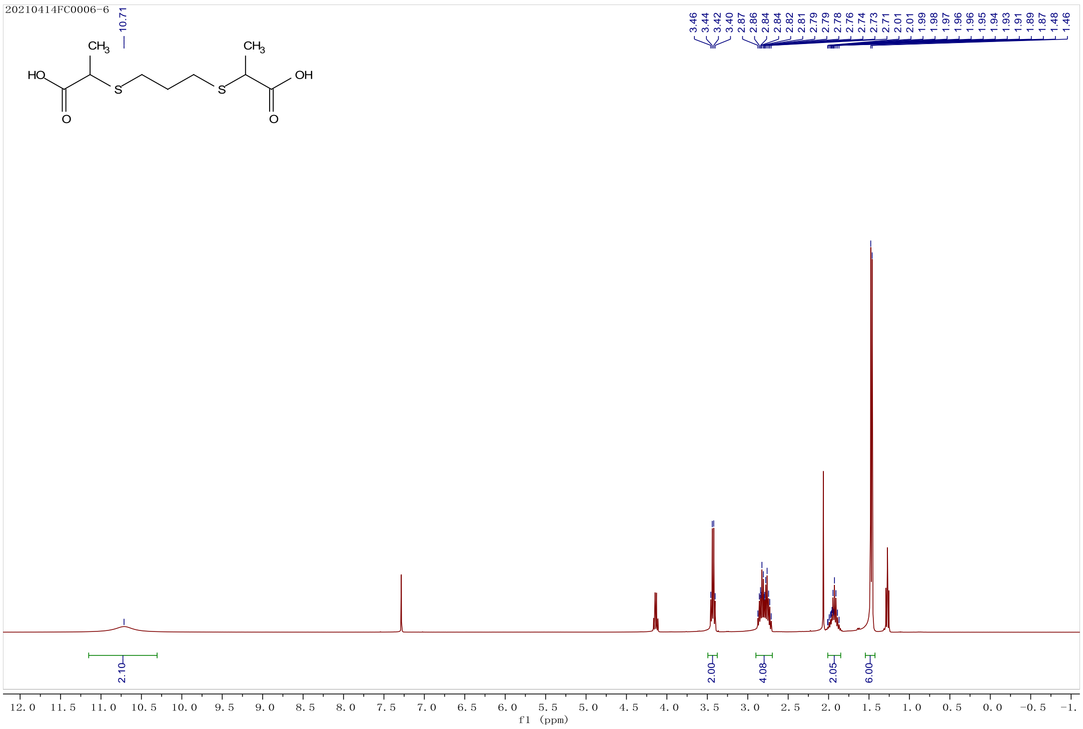

Figure S4. ¹H NMR spectrum of compound **4** ($CDCl_3$)

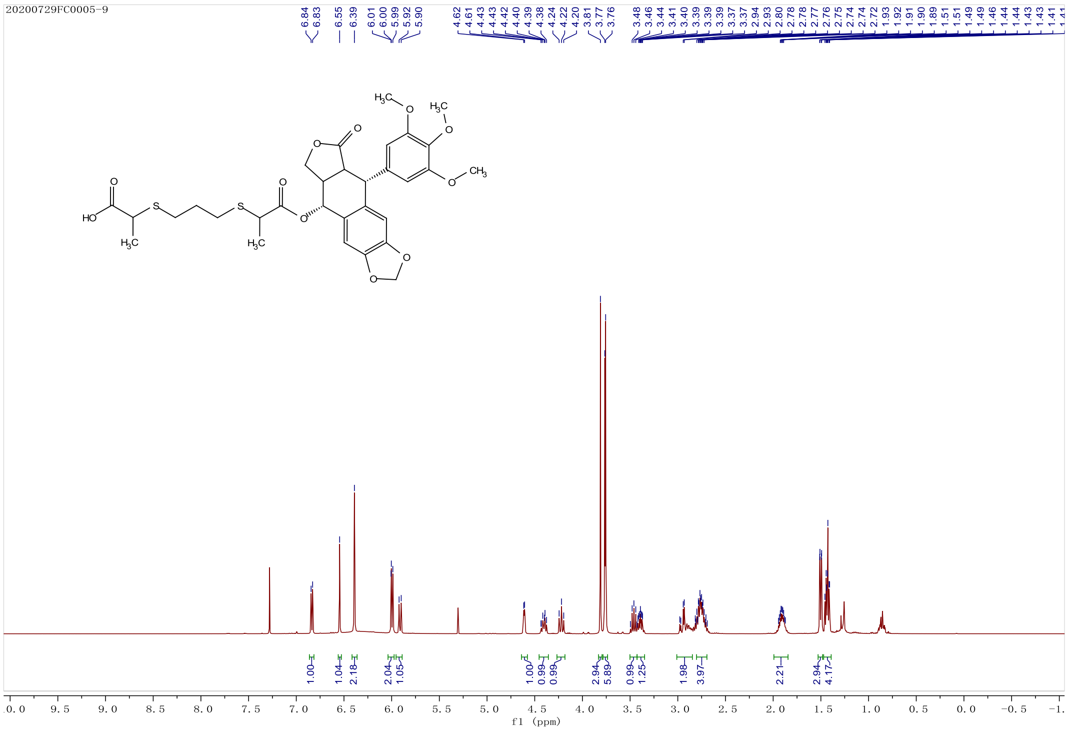

Figure S5. ^{1}H NMR spectrum of compound **5** ($CDCl_3$)

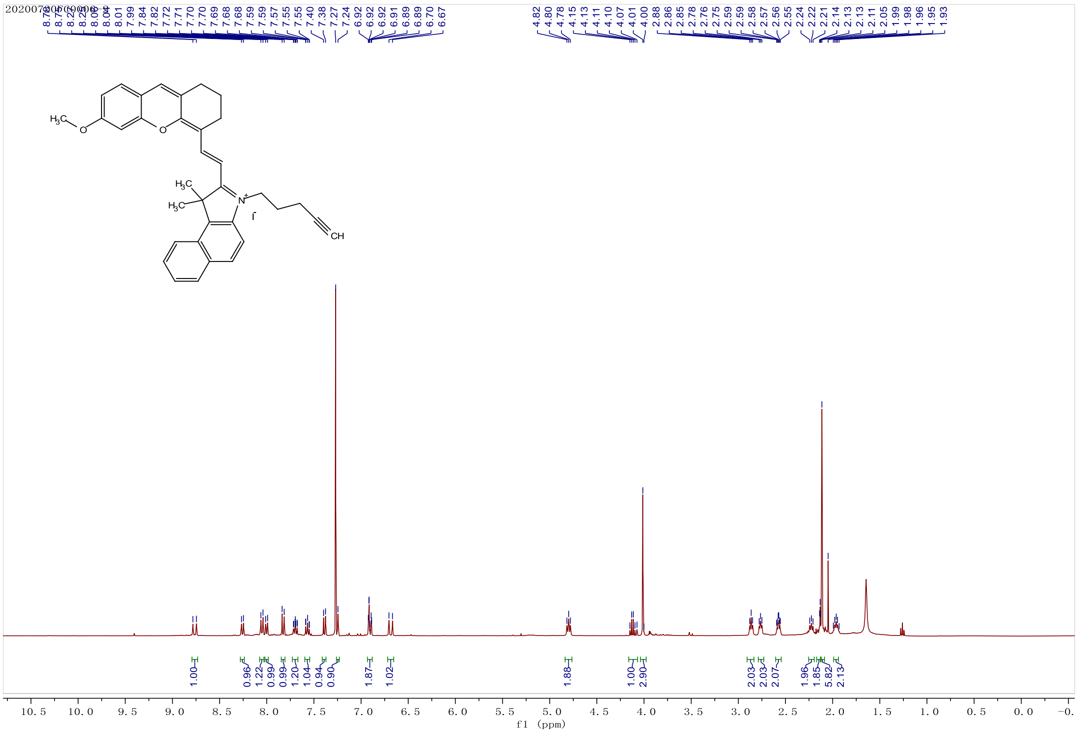

Figure S6. ^{1}H NMR spectrum of compound **12** ($CDCl_3$)

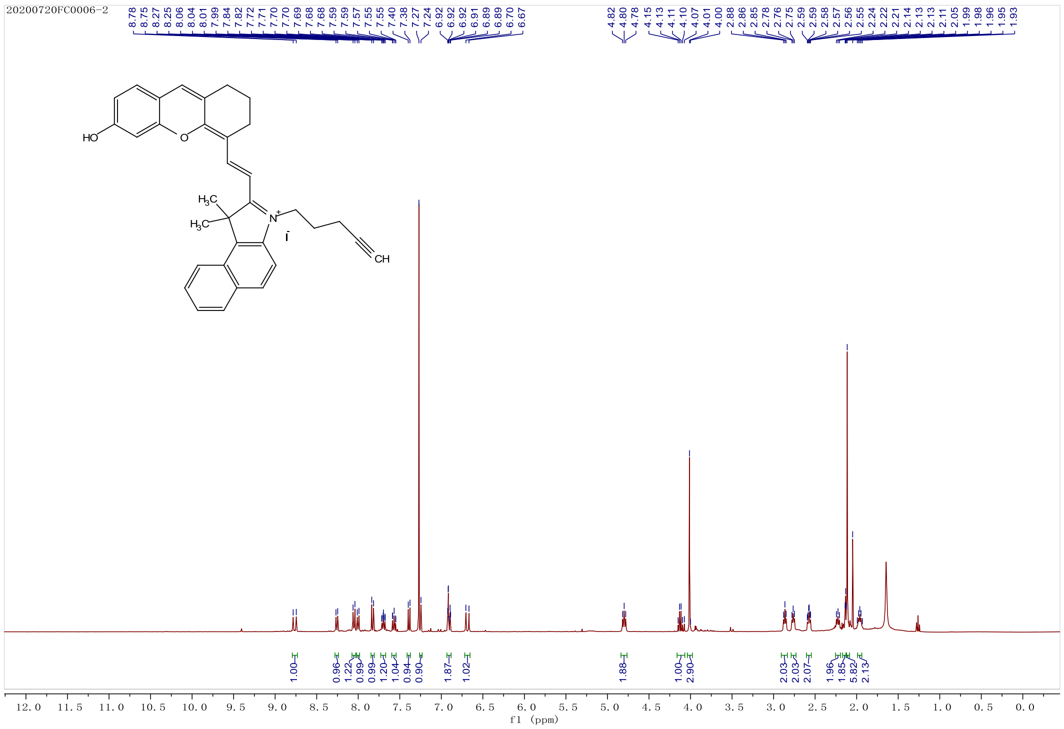

Figure S7. ^{1}H NMR spectra of compound **13**

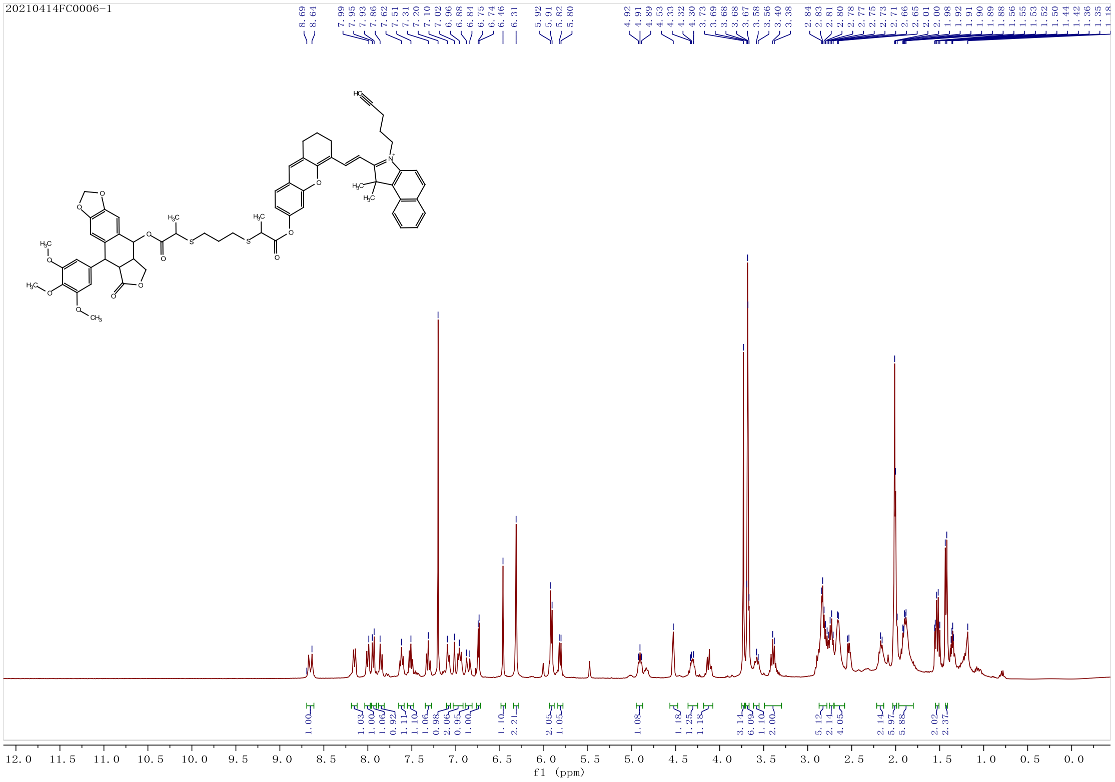

Figure S8. ^{1}H NMR spectra of compound **14**

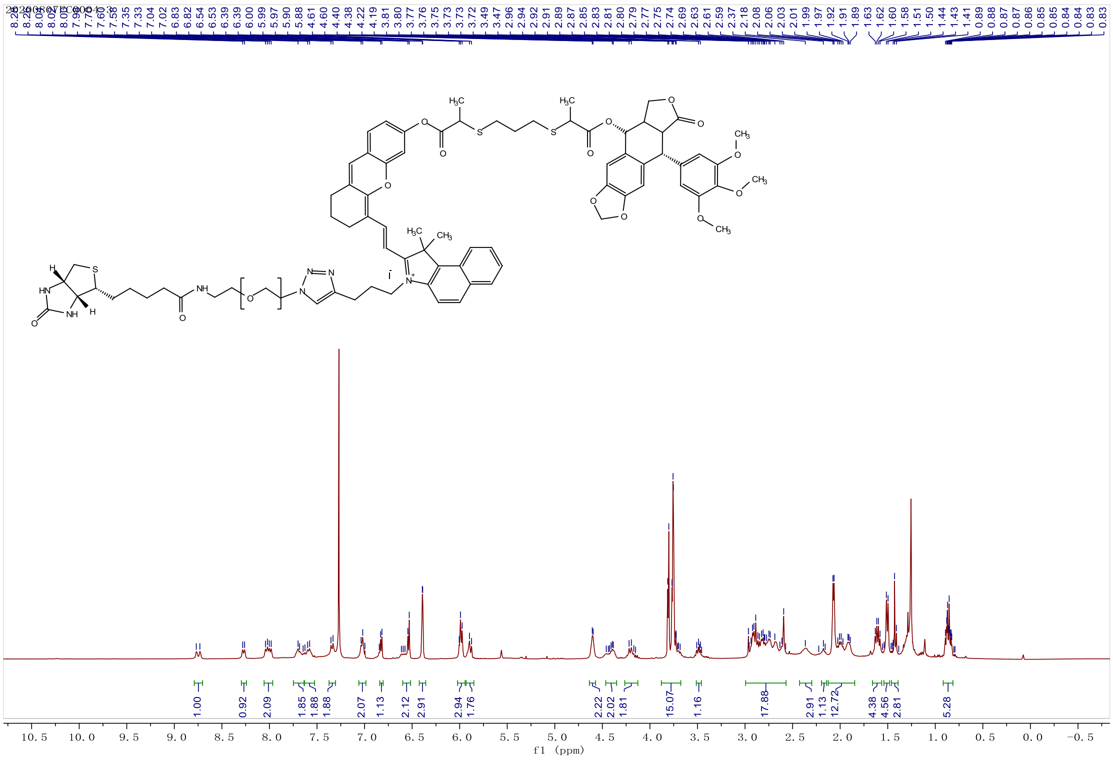

Figure S9. ^{1}H NMR spectra of compound **15**

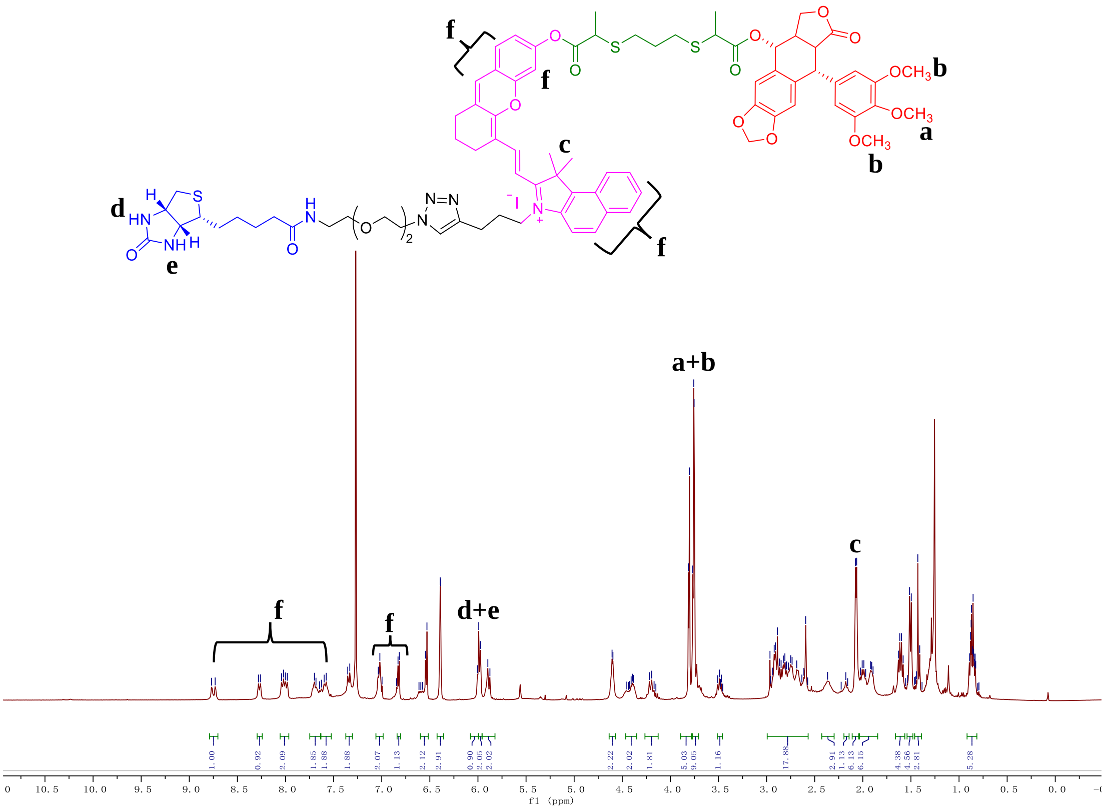

Figure S10. **PBB** is composed of podophyllotoxin, hemicyanine (CyOH), and biotin and their typical peaks of ^{1}H NMR have been displayed respectively. For the region a+b between 3.78 and 3.70 ppm, one peak with an integral of 9 is considered to be the

methoxy of podophyllotoxin. Peak c (δ= 2.07 ppm) with an integral of 6 is attributed to methyl of CyOH. The peaks f attributed to Ar-H of CyOH have been marked in this spectrum. In addition, chemical shift of N-H of carbonyldiimino of biotin is about 6 ppm, which matches the peak well (δ= 5.98 ppm).

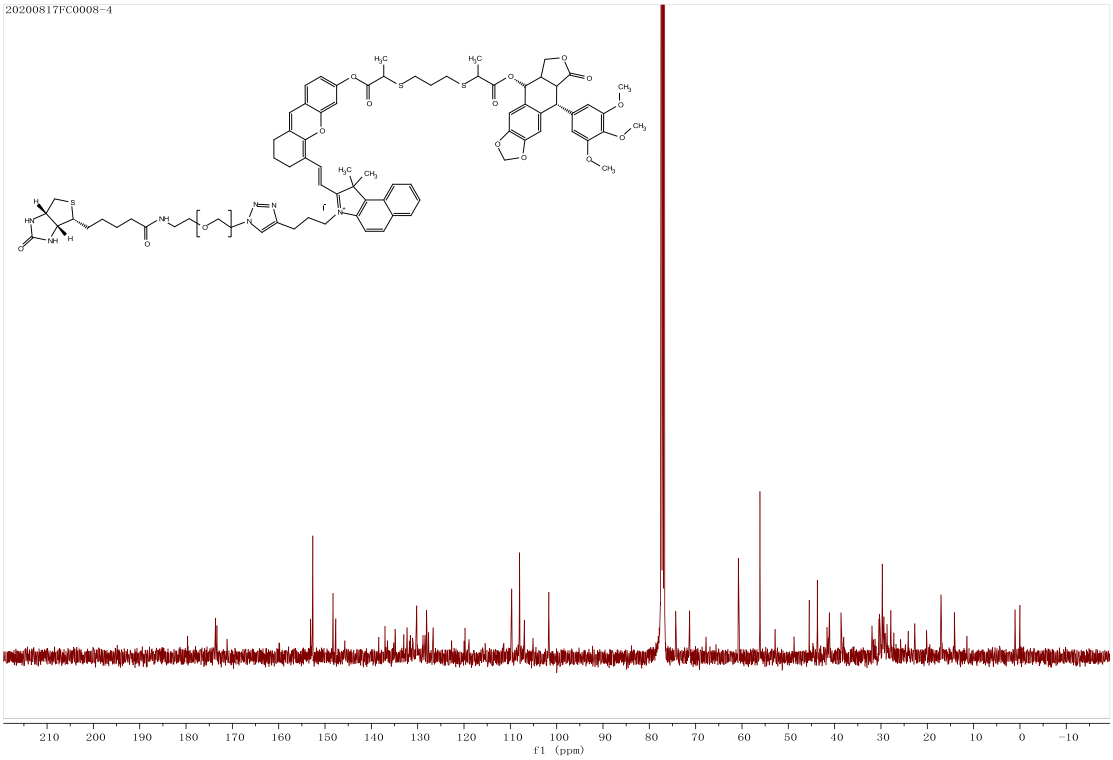

Figure S11. ^{13}C NMR spectra of compound **15**

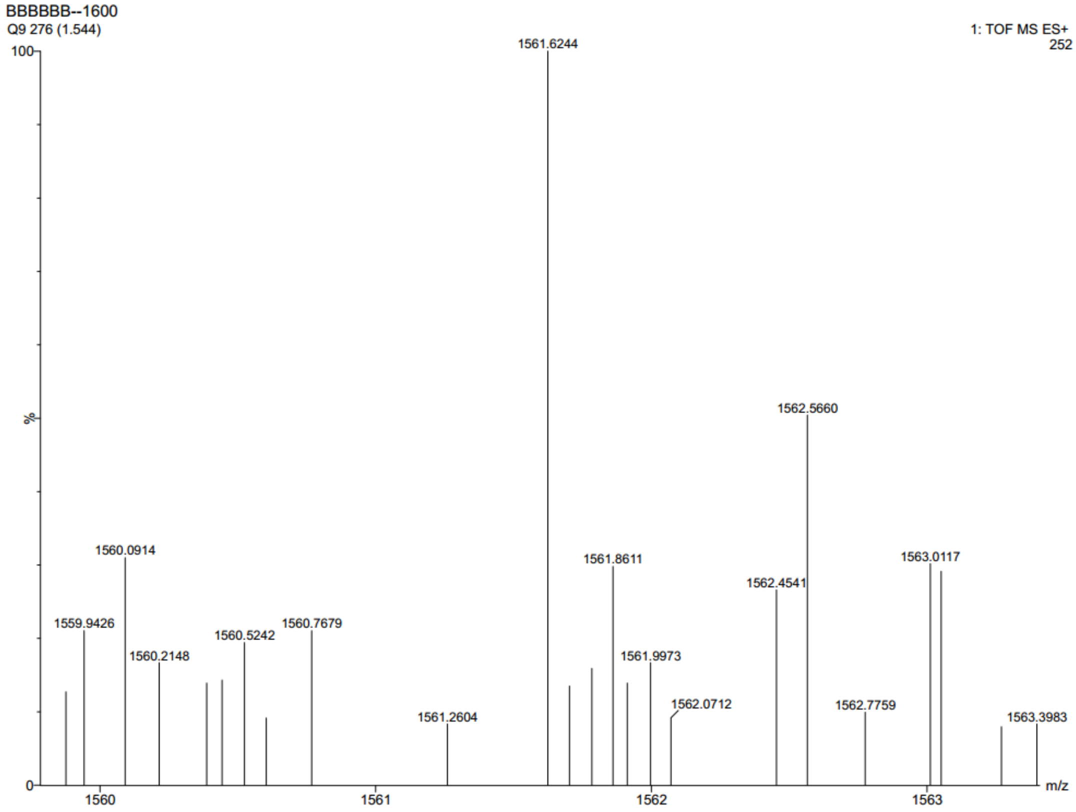

Figure S12. HR-MS spectra of compound **15**

References

1. Sharma A, Lee MG, Won M, Koo S, Arambula JF, Sessler JL, et al. Targeting heterogeneous tumors using a multifunctional molecular prodrug. J Am Chem Soc. 2019; 141: 15611-8.
